# Interoperable Models for Identifying Critically Ill Children at Risk of Neurologic Morbidity

**DOI:** 10.1001/jamanetworkopen.2024.57469

**Published:** 2025-02-04

**Authors:** Christopher M. Horvat, Amie J. Barda, Eddie Perez Claudio, Alicia K. Au, Andrew Bauman, Qingyang Li, Ruoting Li, Neil Munjal, Mark S. Wainwright, Tanupat Boonchalermvichien, Harry Hochheiser, Robert S. B. Clark

**Affiliations:** 1 Department of Critical Care Medicine, University of Pittsburgh, Pittsburgh, Pennsylvania; 2 Safar Center for Resuscitation Research, University of Pittsburgh, Pittsburgh, Pennsylvania; 3 Barda Analytics Consulting LLC, North Royalton, Ohio; 4 Department of Biomedical Informatics, University of Pittsburgh, Pittsburgh, Pennsylvania; 5 Seattle Children’s Hospital, Seattle, Washington; 6 Department of Pediatrics, University of Wisconsin, Madison

## Abstract

**Importance:**

Decreasing mortality in the field of pediatric critical care medicine has shifted practicing clinicians’ attention to preserving patients’ neurodevelopmental potential as a main objective. Earlier identification of critically ill children at risk for incurring neurologic morbidity would facilitate heightened surveillance that could lead to timelier clinical detection, earlier interventions, and preserved neurodevelopmental trajectory.

**Objectives:**

To develop machine-learning models for identifying acquired neurologic morbidity in hospitalized pediatric patients with critical illness and assess correlation with contemporary serum-based, brain injury–derived biomarkers.

**Design, Setting, and Participants:**

This prognostic study used data from all children admitted to a quaternary pediatric intensive care unit in a large, freestanding children’s hospital in Western Pennsylvania between January 1, 2010, and December 31, 2022. External model validation used data from children admitted between January 1, 2018, and December 31, 2023, to a quaternary pediatric intensive care unit in a large, freestanding children’s hospital that serves as a referral center for the 5-state region of Washington, Wyoming, Alaska, Montana, and Idaho.

**Exposures:**

Critical illness.

**Main Outcomes and Measures:**

The outcome was neurologic morbidity, defined according to a computable, composite definition at the development site or an order for neurocritical care consultation at the validation site. Models were developed using varying time windows for temporal feature engineering and varying censored time horizons between the last feature and the identified neurologic morbidity. A generalizable model created at the development site was optimized and assessed at an external validation site. Correlation was assessed between development site model predictions and measurements of brain biomarkers from a convenience cohort.

**Results:**

After exclusions, there were 18 568 encounters from 2010 to 2022 in the development site generalizable model cohort (median age, 70 [IQR, 18-161] months; 8325 [45%] female). There were 6825 encounters from 2018 to 2021 at the external validation site (median age, 96 [IQR 18-171] months; 3159 [46%] female). A generalizable extreme gradient boosted model with a 24-hour time horizon and 48-hour feature engineering window demonstrated an F1 score of 0.37 (95% CI, 0.33-0.40), area under the receiver operating characteristics curve of 0.81 (95% CI, 0.78-0.83), and number needed to alert of 4 at the validation site. After recalibration at the validation site, the Brier score was 0.04. Serum levels of the brain injury biomarker glial fibrillary acidic protein significantly correlated with model output (*r*_s_ = 0.34; *P* = .007).

**Conclusions and Relevance:**

This prognostic study of prediction models for detecting neurologic morbidity in critically ill children demonstrated a well-performing ensemble of models with biomolecular corroboration. Prospective assessment and refinement of biomarker-coupled risk models in pediatric critical illness are warranted.

## Key Points

**Question:**

Can interoperable models for predicting neurologic deterioration in critically ill children be developed, correlated with serum-based brain injury–derived biomarkers, and validated at an external site?

**Findings:**

This prognostic study demonstrated an area under the receiver operating characteristic curve of 0.81 and a number needed to alert of 4. Predictions correlated with levels of glial fibrillary acidic protein in a subset of children.

**Meaning:**

Well-performing prediction models coupled with brain biomarkers may help identify critically ill children at risk for acquired neurologic morbidity.

## Teaser

This decision analytic model study evaluates machine-learning models for identifying critically ill children at high risk for acquired neurologic morbidity and assesses correlation with contemporary serum-based, brain injury–derived biomarkers.

## Introduction

An estimated 340 000 children are hospitalized with critical illness every year in the US, and brain injury has been cited as the proximate cause of death in approximately 90% of previously healthy children who do not survive their intensive care admission.^1,2^ Of children who survive critical illness, acquired neurologic morbidity can have long-lasting implications, which range from mild impairments in cognition to profound debilitation. Decreasing mortality in the field of pediatric critical care has led to increased attention on longer-term functional outcomes of children who survive an intensive care admission.^3^

Granular time series data harbored in the electronic health record (EHR) offer a rich training ground for probabilistic models of important patient outcomes. Implementing well-performing models as clinical decision support systems is a promising approach for improving outcomes related to many different conditions and situations, although there are currently no established tools for identifying children at risk for new brain injury.^4,5,6,7^ Recently enacted federal mandates in the US are promoting the development of EHRs that facilitate the deployment of interoperable decision support tools built to leverage a core dataset.^8^

The main objective of this study was to construct and externally validate predictive models to support the identification of critically ill children at high risk for acquired neurologic morbidity, as a first step toward the development of a decision support tool that might be used to forewarn of neurologic morbidity among critically ill children and to aid in the enrichment of prospective trials examining strategies to mitigate the risk of brain injury during pediatric critical illness. A second objective was to corroborate the biological underpinnings of the developed prediction models by assessing correlation with novel, brain injury–derived, serum-based biomarkers of brain injury obtained from a diagnostically diverse cohort of critically ill children.

## Methods

### Study Sites

This prognostic study used data from all children admitted to a quaternary pediatric intensive care unit (PICU) in a large, freestanding children’s hospital between January 1, 2010, and December 31, 2022. The development site PICU serves a region of approximately 5 million people, encompassing Western Pennsylvania and bordering states, and is a level I pediatric trauma center. External model validation occurred using data from children admitted between January 1, 2018, and December 31, 2023, to a quaternary PICU in a large, freestanding children’s hospital that serves as a referral center for the 5-state region of Washington, Wyoming, Alaska, Montana, and Idaho. Race and ethnicity were not collected or reported for model development because this information was not considered necessary. Approval was granted by the institutional review boards of the University of Pittsburgh and Seattle Children’s Hospital. Findings are reported according to the Transparent Reporting of a Multivariable Prediction Model for Individual Prognosis or Diagnosis (TRIPOD) reporting guideline.

### Model Development Frameworks

Conceptualization of the model adhered to the Littenberg framework for the development of clinical decision support tools, which considers the clinical and technical plausibility of the tool, as well as the process outcomes, patient outcomes, and eventual societal outcomes addressed by the tool (eTable 1 in the Supplement 1).^9^ The first 5 steps of the Cross-Industry Standard Process for Data Mining (CRISP-DM) framework were followed for model design. CRISP-DM outlines 6 steps for data science projects: (1) understanding the use case, (2) understanding the data, (3) data curation, (4) model development, (5) model evaluation, and (6) model deployment.^10^

### Model Development Approach

Model development proceeded in 2 phases: (1) development of models for use locally at the development site constructed from 45 structured EHR data elements and (2) creation of generalizable models at the development site using 41 variables that were also available at the external validation site for model assessment. Data from the external validation site were not used to train any model. The outcome of neurologic morbidity was defined using structured EHR data surrogates based on each study site’s clinical and electronic workflows. At the development site, the outcome was a previously validated, computable, composite definition of neurologic morbidity that incorporated orders for electroencephalography, brain computed tomography, brain magnetic resonance imaging, or indicators of treated delirium within 72 hours of one another (eTable 2 in Supplement 1).^11^ This outcome has also been validated in a separate cohort of children with sepsis.^12^ At the validation site, orders for a neurocritical care service consultation were deemed to be the most reliable surrogate for neurologic morbidity during an episode of critical illness. Data for control cases (hospitalized children who did not meet the definition of a neurologic morbidity) were collected from a random period during the encounter with preference for a window following the first PICU admission.

Candidate data elements for model construction were selected based on clinical expertise and with attention to the US Core Data for Interoperability (USCDI) requirements to facilitate eventual, interoperable deployment (eTables 3 and 4 in Supplement 1).^13^ A BRAIN A-I (Biodigital Rapid Alert to Identify Neurologic Morbidity, A-I bundle) standard clinical vocabulary value set was filed with the National Library of Medicine’s Value Set Authority Center.^14^ Features were engineered with the dual aims of representing the temporality of the data while also preserving clinical interpretability of the features, using previously reported methods.^15^ Features were then discretized, or categorized into information bins, with missingness encoded as a feature. In addition to preserving possible information associated with missingness, discretization was performed to reduce the influence of outlier data, represent data nonlinearity in linear modeling processes such as logistic regression, further mitigate overfitting, and preserve clinical interpretability of the features. Data from 2010 to 2019 were divided 3:1 into development and initial validation sets, and data from 2020 to 2022 were reserved as a holdout test dataset for the final best-performing models. Additional details of data curation and model development are in the eMethods in Supplement 1. eTable 5 and eFigures 1 to 3 in Supplement 1 summarize model construction at the development site and evaluation at the external validation site. At the development site, data were queried from an Oracle (Oracle Corp) data warehouse containing a subset of transformed tables from the Cerner Millennium database (Oracle Cerner). The model was developed and assessed using Python, version 3.10.11 (Python Software Foundation), Jupyter, version 1.0.0 (Project Jupyter), and the packages pandas, version 1.5.3; NumPy, version 1.25.0; Matplotlib, version 3.71.1; Sklearn, version 1.1.1; XGBoost, version 1.7.3; Seaborn, version 0.11.2; SHAP, version 0.41.0; and tqdm, version 4.65.0.

### Biomolecular Corroboration of the Model at the Development Site

Model predictions were compared with measured levels of 6 serum-based, brain injury–derived biomarkers of brain injury obtained from a previously assembled convenience cohort of 101 children hospitalized between 2012 and 2014. The biomarkers were ubiquitin C-terminal hydrolase-L1, glial fibrillary acidic protein (GFAP), myelin basic protein, neuron-specific enolase, S100 calcium-binding protein B, and spectrin breakdown product 150. After prospective consent from a legal guardian, biomarker levels were collected for up to 7 consecutive days from critically ill children with preexisting central venous catheters or arterial catheters. Details of the assays are provided in the eMethods in Supplement 1. Maximum values of each biomarker for each encounter were assessed for correlation with the predicted probability of neurologic deterioration for that encounter. Patients were determined to have a neurologic complication by EHR review if it occurred no more than 7 days after the last date a biomarker was collected.

### Statistical Analysis

The top-performing model was selected based on F1 score, considering a clinically actionable time horizon, which is the period between the most recent data available to the model and the outcome of interest, as well as the volume of available training data for feature engineering. Models with a difference of less than 0.15 in F1 scores were then compared both by visual inspection of calibration plots and Brier scores. Additional Fβ thresholds of 0.5, 2.0, and 3.0 were secondarily evaluated to identify whether there were any substantial differences in the optimal classifier based on the relative weight of recall compared with precision. Statistical performances of the top-performing models were evaluated at varied model prediction thresholds ranging from 0.025 to 0.9, with 0.025 set to determine model performance in a hypothetical scenario in which sensitivity is a greater priority than positive predictive value. Spline regression was performed on top-performing models to improve calibration.^16,17^ Normally distributed continuous data are presented as means and 95% CIs, nonnormally distributed continuous data are presented as medians with IQRs, and categorical data are presented as numbers with corresponding percentages. Model discrimination was compared with the discrimination of the last Glasgow Coma Scale (GCS) score before the censored time horizon using the method of DeLong et al.^18^ For the biomolecular corroboration analysis, Spearman rank order correlation was assessed between a chart-adjudicated neurologic morbidity outcome and the composite neurologic morbidity outcome, as well as between the probability output of top-performing models and the composite neurologic morbidity outcome. Correlation was then assessed between biomarker levels and the probability output of the top-performing models. Notched boxplots with overlying violin plots were constructed for significantly correlated biomarkers by dichotomizing predicted neurologic morbidity according to whether the probability was <0.5 or ≥0.5. The distributions of biomarker measurements were normalized for plotting using log transformation, and significance testing was assessed using an independent *t* test. An α < .05 was considered statistically significant. Statistical analyses not performed in Python were performed in R software, version 4.3.1 (R Foundation).

## Results

### Development Site Models Performance

**Figure 1.**
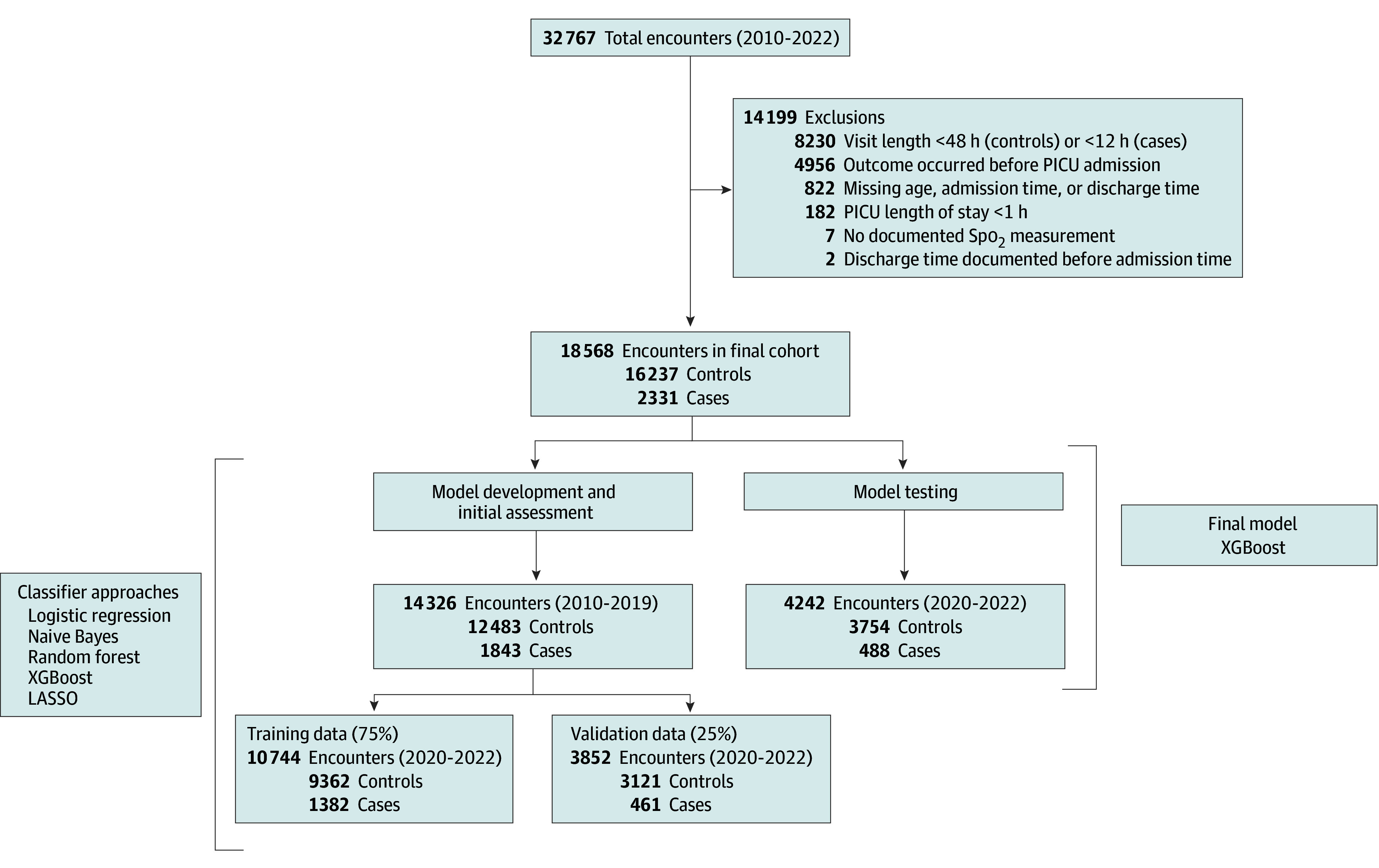
Cohort Ascertainment for the Final Model at the Development Site The model was tested using data from 2020 to 2022. The final model included features engineered using 48 hours of preceding data and censoring 12 hours before the event for cases. Initial model development and validation proceeded using data from 2010 to 2019. LASSO indicates least absolute shrinkage and selection operator; PICU, pediatric intensive care unit; Spo_2_, oxygen saturation as measured by pulse oximetry; XGBoost, extreme gradient boosting.

After exclusions, cohort sizes ranged from 14 222 to 23 873 encounters, with 18 568 encounters in the final model cohort (median age, 70 [IQR, 18-161] years; 10 243 [55%] male and 8325 [45%] female) (eTable 6 in Supplement 1; Figure 1). Patients were slightly older, received less mechanical ventilation, and received fewer sedative-analgesic medications in the final test dataset compared with the training and validation datasets (Table 1). The final model evaluated in the test dataset was the extreme gradient boosting (XGBoost) model with a 12-hour time horizon and 48-hour feature window. This model was determined by investigator agreement to be a reasonable balance of favorable F1 scores, calibration as assessed by a Brier score, visual inspection of the calibration plot, clinically actionable time horizon, and sufficient cohort size for the training, validation, and test datasets. Complete development site model performance characteristics, 1 for each of the combinations of a 6-, 12-, and 24-hour censored time horizons and 24-, 48-, and 72-hour feature windows selected based on F1 score, are detailed in eTables 7 to 9 in Supplement 1. Each approach generated 605 features before information gain feature selection. The F1 scores are reported in eTable 10 in Supplement 1. Additional Fβ scores largely agreed with the model assessments provided by F1 scores and are presented in eTable 11 in Supplement 1.

**Table 1.** Demographic Characteristics of the Study Participants by Site and Dataset

Characteristic	No. (%) of participants^a^				
	Development site				Validation site, generalizable model validation dataset (n = 6825)
	Entire cohort (N = 18 568)	Training dataset (n = 10 744)	Validation dataset (n = 3582)	Final test dataset (n = 4242)	
Age, median (IQR), mo	70 (18-161)	67 (18-158)	68.5 (18-160)	77 (19-167)	96 (18-171)
Sex					
Female	8325 (45)	4748 (44)	1593 (44)	1984 (47)	3159 (46)
Male	10 243 (55)	5996 (56)	1989 (56)	2258 (53)	3666 (54)
Glasgow Coma Scale score, median (IQR)^b^	15 (12-15)	15 (11-15)	15 (11-15)	15 (14-15)	14 (14-15)
Mechanical ventilatory support	5352 (29)	3322 (31)	1143 (32)	887 (21)	1948 (29)
Endotracheal tube	1759 (9)	1142 (11)	370 (10)	247 (6)	1283 (19)
Vasoactive medication^c^	720 (4)	393 (4)	148 (4)	179 (4)	221 (3)
Sedative-analgesic medication^d^	4660 (25)	2948 (27)	987 (27)	727 (17)	1930 (28)

^a^ Unless otherwise indicated.

^b^ The last recorded Glasgow Coma Scale score for the encounter before the censored time horizon.

^c^ Vasoactive medications include dobutamine, dopamine, epinephrine, norepinephrine, and milrinone.

^d^ Sedative-analgesic medications include fentanyl, hydromorphone, midazolam, and morphine.

**Figure 2.**
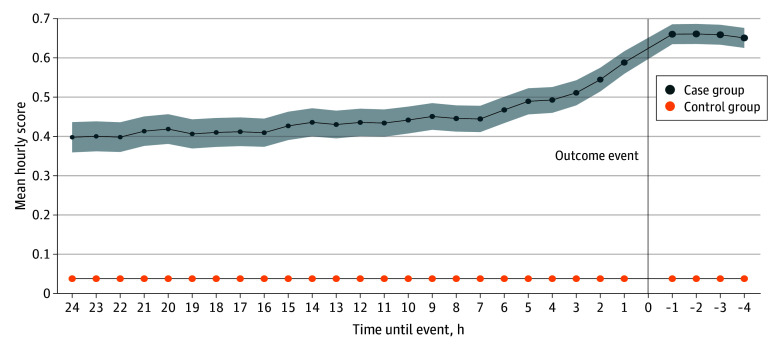
Mean Hourly Scores in the Test Dataset (Encounters With a PICU Stay in 2020 to 2022) for the Extreme Gradient Boosted Model Developed Using Varied Time Horizons (Hours Until Event) and 48-Hour Feature Window The blue dots are the mean hourly scores 24 hours before an event and 4 hours after an event for the case encounters (encounters with an identified neurologic morbidity), and the shaded region represents the 95% CIs. The orange dots are the mean hourly scores for the control encounters (encounters without an identified neurologic morbidity). The CIs for the control encounters are not discernible in the figure due to the large cohort size. The size of the dots is proportionate to the cohort size at that time point. PICU indicates pediatric intensive care unit.

The final model contained 352 features and had a number needed to alert (NNA) of 2 when considering a model prediction of greater than or equal to 0.5 as positive. At a model prediction threshold of 0.025 in the test dataset, sensitivity increased to 0.86, and the NNA was 4. Statistical performance of the top-performing validation models and final test model at a range of output thresholds is given in eTable 12 and eFigure 4 in Supplement 1. All development site models had an NNA of 2 to 3 at a prediction threshold of 0.1. In the test dataset, the final model’s predictions had a sensitivity of 0.47 (range for all models in the validation dataset, 0.24-0.63), specificity of 0.98 (range, 0.96-0.99), area under the precision recall curve (AUPRC) of 0.68 (range, 0.39-0.78), and area under the receiver operating characteristic curve (AUROC) of 0.89 (range, 0.80-0.87). The final model had significantly greater discrimination compared with the last GCS AUROC of 0.72 obtained before the censored time horizon (*P* < .001). Calibration plots of models with comparable performance based on F1 scores are displayed in eFigure 5 in Supplement 1. The top 10 features of the final model are displayed in eFigure 6 in Supplement 1. Mean hourly scores for cases and controls at varied time horizons for the 24 hours before and 4 hours after an outcome event are displayed in Figure 2.

### Biomolecular Corroboration at the Development Site

**Figure 3.**
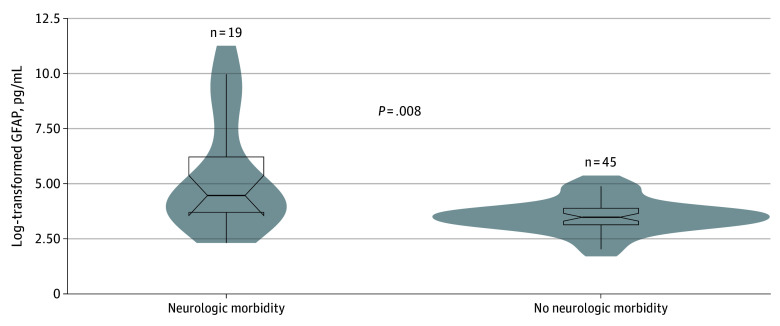
Log-Transformed Maximum Glial Fibrillary Acidic Protein (GFAP) Measurements for a Convenience Cohort of 64 Patients, Stratified by Predicted Neurologic Morbidity Using the 12-Hour Time Horizon 48-Hour Feature Window Extreme Gradient Boosting Model

Of the 101 patients with available brain injury–derived biomarkers measured, 64 also had model predictions for the 12-hour time horizon and 48-hour feature window models. The EHR-adjudicated neuromorbidity within 7 days of the last biomarker collection was significantly correlated with the composite neurologic morbidity outcome (*r*_s_ = 0.38; *P* = .002). Extreme gradient boosting predictions were significantly correlated with maximum GFAP measurements (*r*_s_ = 0.34; *P* = .007) (Figure 3).

### Generalizable Model Performance

There were 6825 encounters in the external validation site final cohort (387 cases and 6438 controls; median [IQR] age, 96 [18-171] months; 3666 [54%] male and 3159 [46%] female). Cohort ascertainment for the generalizable model is reported for the 24-hour time horizon and 48-hour feature window model at the development site in eTable 6 in Supplement 1 and for the validation site in eTable 13 in Supplement 1. The generalizable model performance at the development and validation sites is reported in Table 2. Performance was comparable to earlier 24-hour time horizon 48-hour feature window models at the development site. Because the XGBoost and logistic regression models performed comparably, both were assessed at the validation site. As assessed by an F1 score of 0.37 (95% CI, 0.33-0.40) at a threshold of 0.5, the top-performing model was the XGBoost model, with an external validation AUROC of 0.81 (95% CI, 0.78-0.83), AUPRC of 0.51 (95% CI, 0.46-0.56), and NNA of 4. Model performance characteristics across varied thresholds are given in eTable 14 and eFigure 7 in Supplement 1. Calibration was again excellent at the development site, initially poor at the external validation site, then substantially improved after spline recalibration at the validation site (eFigure 8 in Supplement 1). Feature importance analysis for the generalizable model was similar between the development and validation sites (eFigure 9 in Supplement 1). All models outperformed the GCS.

**Table 2.** Performance of the Generalizable Model at the Development and External Validation Sites

Variable	Value (95% CI)							
	Development (10 457 encounters [1095 cases and 9362 controls])		Validation (3486 encounters [365 cases and 3121 controls])		Test (4152 encounters [398 cases and 3754 controls])		External validation (6825 encounters [387 cases and 6438 controls])	
Model	XGB	LR	XGB	LR	XGB	LR	XGB	LR
Feature selection	IG	IG	IG	IG	IG	IG	IG	IG
AUROC	1.00 (1.00-1.00)	0.90 (0.89-0.91)	0.81 (0.79-0.83)	0.81 (0.80-0.83)	0.87 (0.85-0.89)	0.86 (0.84-0.87)	0.81 (0.78-0.83)	0.82 (0.79-0.84)
AUPRC	0.99 (0.99-1.00)	0.71 (0.70-0.72)	0.52 (0.47-0.54)	0.54 (0.51-0.56)	0.62 (0.58-0.63)	0.61 (0.59-0.66)	0.51 (0.46-0.56)	0.48 (0.42-0.53)
PPV, %	1.00 (1.00-1.00)	0.84 (0.82-0.86)	0.67 (0.65-0.70)	0.69 (0.65-0.73)	0.82 (0.76-0.87)	0.80 (0.77-0.87)	0.26 (0.24-0.29)	0.19 (0.17-0.21)
NPV, %	0.99 (0.99-0.99)	0.94 (0.94-0.95)	0.93 (0.92-0.93)	0.93 (0.93-0.95)	0.94 (0.93-0.94)	0.94 (0.93-0.94)	0.97 (0.97-0.98)	0.98 (0.97-0.98)
Sensitivity, %	0.91 (0.89-0.92)	0.47 (0.45-0.51)	0.35 (0.31-0.38)	0.41 (0.40-0.47)	0.35 (0.32-0.36)	0.38 (0.35-0.43)	0.61 (0.56-0.66)	0.70 (0.66-0.75)
Specificity, %	1.00 (1.00-1.00)	0.99 (0.99-0.99)	0.98 (0.98-0.98)	0.98 (0.97-0.98)	0.99 (0.99-0.99)	0.99 (0.99-0.99)	0.90 (0.89-0.90)	0.81 (0.80-0.82)
F1 score	0.95 (0.94-0.96)	0.61 (0.59-0.63)	0.46 (0.43-0.49)	0.51 (0.50-0.55)	0.49 (0.46-0.50)	0.52 (0.49-0.57)	0.37 (0.33-0.40)	0.29 (0.27-0.32)
F2 score	0.92 (0.91-0.93)	0.52 (0.49-0.55)	0.39 (0.35-0.42)	0.45 (0.44-0.50)	0.39 (0.37-0.40)	0.43 (0.40-0.48)	0.48 (0.45-0.52)	0.45 (0.42-0.48)
F3 score	0.92 (0.90-0.93)	0.50 (0.47-0.53)	0.37 (0.33-0.40)	0.43 (0.42-0.49)	0.37 (0.34-0.38)	0.41 (0.37-0.45)	0.54 (0.50-0.58)	0.55 (0.51-0.59)
F0.5 score	0.98 (0.98-0.98)	0.73 (0.71-0.75)	0.57 (0.54-0.60)	0.60 (0.58-0.64)	0.65 (0.61-0.67)	0.66 (0.63-0.72)	0.30 (0.27-0.33)	0.22 (0.20-0.24)

Abbreviations: AUPRC, area under the precision recall curve; AUROC, area under the receiver operating characteristics curve; IG, information gain; LR, logistic regression; NPV, negative predictive value; PPV, positive predictive value; XGB, XGBoost (extreme gradient boosting).

## Discussion

In this study, we constructed well-performing models for predicting neurologic morbidity among critically ill children using EHR data from 2 large children’s hospitals. A generalizable model demonstrated robust performance at both the development and external validation sites. All models outperformed the GCS, supporting machine learning–based methods to facilitate clinical activities, including identification of high-risk patients for clinical intervention and identification of an enriched population for enrollment in clinical trials.^19^ The generalizable model relies on 41 variables, 37 of which are included in USCDI versions 1 or 2 and are therefore expected to ease eventual work associated with deployment. By largely adhering to data elements prioritized by the USCDI, the developed models have a clearer path to implementation in modern informatics architectures capable of data transfer using standard clinical vocabularies and the fast health care interoperability resources standard.^20^

Many predictive models are constructed using a snapshot of information from a discrete moment in time.^21,22^ The performance of the current models was likely bolstered by incorporating features engineered using vector space representations of patient state, resulting in performance metrics that surpass those of other commonly used critical care risk models.^15^ The Simplified Acute Physiology Score, a commonly used mortality prediction tool for critically ill adults, has reported AUPRCs of 0.2 to 0.3 for in-hospital and 30-day mortality.^23^ The Sequential Organ Failure Assessment, quick Sequential Organ Failure Assessment, and systemic inflammatory response syndrome criteria have reported AUPRCs of 0.06, 0.1, and 0.09, respectively, for predicting mortality at the time of sepsis onset.^24^ By comparison, our model ensemble had AUPRCs ranging from 0.39 to 0.78 at the development site and 0.2 to 0.42 at the validation site.

Correlation between a top-performing model and measurements of GFAP from a convenience cohort is compatible with a previous investigation of brain biomarkers in critically ill children.^25^ GFAP is found in astrocytes and plays a role responding to central nervous system injuries and related neurodegeneration.^26^ GFAP measurements from our convenience cohort were obtained for the first 7 days of the PICU stay and may have been obtained remotely from an incurred brain injury, including one detected by the composite neurologic morbidity outcome. Notably, our composite neurologic morbidity outcome was significantly correlated with EHR-adjudicated neurologic morbidity, and an XGBoost model’s predictions were significantly associated with GFAP levels. Most extensively studied in the context of traumatic brain injury, GFAP may be useful for identifying more subtle insults to the central nervous system, and the ability to measure GFAP in the bloodstream in nontraumatic diseases might relate to its dispersion into the bloodstream via recently discovered glymphatic pathways.^27,28^ Our models may prove useful for determining which patients should have a GFAP level obtained and also may be coupled with the GFAP measurements to bolster model performance.

### Limitations

This work has some important limitations. The development site test dataset included slightly different population characteristics compared with the development and validation datasets, likely owing to COVID-19 pandemic–related changes in case mix in the test dataset. This difference may have contributed to improved performance of the generalizable models in the test dataset compared with the validation dataset. Use of a composite definition of neurologic morbidity intrinsically omits occult neurologic morbidities that did not trigger clinical action and represents a source of potential bias in model development. Although the computable composite definition of neurologic morbidity used in the current study has previously demonstrated high specificity, the modest sensitivity of the definition suggests that the current models may miss neurologic morbidities that do not warrant inpatient imaging, electroencephalography, a mental health assessment, or a medication directed at psychosis or delirium. This limitation, however, can be mitigated by assessing performance characteristics, including varied Fβ scores or sensitivities at different output thresholds, according to context and adjusting the model actionable threshold in a manner tailored to the clinical environment in which it is deployed. Statistical metrics deteriorated at the validation site compared with those observed in the test dataset at the development site. Notably, the GCS had a lower AUROC at the validation site compared with the development site, suggesting that the choice of neurocritical care consult as an outcome influenced performance characteristics.

## Conclusions

In this prognostic study, we developed well-performing models for predicting which children with critical illness were at risk for neurologic morbidity. A flexible, distributed strategy for model development in partnership with an external validation site demonstrated the utility of adapting to varied informatics infrastructures and EHR deployments to generate well-performing predictive models for a common clinical goal. A generalizable model demonstrated robust performance in external validation. Prospective, multisite assessment of a generalizable model coupled with brain injury–based biomarkers is warranted to assess the combined utility for identifying patients at high risk for incurred neurologic morbidity and evaluating interventions to improve outcomes in this population.

## Notes

Supplement 1.
eMethods.
**eTable 1.** Littenberg framework **eTable 2.** Data curation steps for individual data elements for the BRAIN AI outcome **eTable 3.** Data curation steps for individual data elements for BRAIN A-I **eTable 4.** Standard vocabulary crosswalk for BRAIN-AI components **eTable 5.** Temporal features and their associated definitions **eTable 6.** Cohort ascertainment and exclusions for varied censored time horizons and feature windows at the development site **eTable 7.** Predictive performance of the XGBoost model with a 12-hour censor horizon and 48-hour feature window in the validation dataset, after manual tuning, and after Bayesian tuning **eTable 8.** Predictive performance of the XGBoost model with a 12-hour censor horizon and 48-hour feature window in the test dataset, after manual tuning, and after Bayesian tuning **eTable 9.** Performance of the optimal models in the validation dataset at the development site **eTable 10.** F1 scores of top performing models in the development site validation dataset **eTable 11.** Fβ scores **eTable 12.** Statistical performance of the 12-hour time horizon, 48-hour feature window XGBoost and logistic regression models **eTable 13.** Cohort ascertainment and exclusions for varied feature windows for the validation site **eTable 14.** Statistical performance of the extreme gradient boosting (XGBoost) and logistic regression generalizable models **eFigure 1.** The process of BRAIN A-I model development and external validation **eFigure 2.** A representation of the time window and censor horizons used to define cases and controls as part of the development, validation, and test cohorts **eFigure 3.** Data cleaning and feature engineering process **eFigure 4.** Plots of key statistical performance metrics **eFigure 5.** Calibration plots and associated Brier scores for the top performing models for varied time horizons and features windows in the validation dataset **eFigure 6.** Top 10 biomarker feature categories **eFigure 7.** Plots of key statistical performance metrics for generalizable model performance **eFigure 8.** Calibration plots for the generalizable model **eFigure 9.** Feature importance analysis for the generalizable model

Supplement 2.
Data Sharing Statement
