# Supplementary material for: Interoperable Models for Identifying Critically Ill Children at Risk of Neurologic Morbidity
Source: JAMA Netw Open. 2025 Feb 4;8(2):e2457469. doi: 10.1001/jamanetworkopen.2024.57469 (PMC11795326; doi:10.1001/jamanetworkopen.2024.57469)
Supplement: Supplement 1. — eMethods. eTable 1. Littenberg framework eTable 2. Data curation steps for individual data elements for the BRAIN AI outcome eTable 3. Data curation steps for individual data elements for BRAIN A-I eTable 4. Standard vocabulary crosswalk for BRAIN-AI components eTable 5. Temporal features and their associated definitions eTable 6. Cohort ascertainment and exclusions for varied censored time horizons and feature windows at the development site eTable 7. Predictive performance of the XGBoost model with a 12-hour censor horizon and 48-hour feature window in the validation dataset, after manual tuning, and after Bayesian tuning eTable 8. Predictive performance of the XGBoost model with a 12-hour censor horizon and 48-hour feature window in the test dataset, after manual tuning, and after Bayesian tuning eTable 9. Performance of the optimal models in the validation dataset at the development site eTable 10. F1 scores of top performing models in the development site validation dataset eTable 11. Fβ scores eTable 12. Statistical performance of the 12-hour time horizon, 48-hour feature window XGBoost and logistic regression models eTable 13. Cohort ascertainment and exclusions for varied feature windows for the validation site eTable 14. Statistical performance of the extreme gradient boosting (XGBoost) and logistic regression generalizable models eFigure 1. The process of BRAIN A-I model development and external validation eFigure 2. A representation of the time window and censor horizons used to define cases and controls as part of the development, validation, and test cohorts eFigure 3. Data cleaning and feature engineering process eFigure 4. Plots of key statistical performance metrics eFigure 5. Calibration plots and associated Brier scores for the top performing models for varied time horizons and features windows in the validation dataset eFigure 6. Top 10 biomarker feature categories eFigure 7. Plots of key statistical performance metrics for generalizable model per [file jamanetwopen-e2457469-s001.pdf]

Supplemental Online Content

Horvat CM, Barda AJ, Perez Claudio E, et al. Interoperable models for identifying critically ill children at risk of neurologic morbidity. *JAMA Netw Open*. 2025;8(2):e2457469. doi:10.1001/jamanetworkopen.2024.57469

eMethods.

eTable 1. Littenberg framework

eTable 2. Data curation steps for individual data elements for the BRAIN AI outcome

eTable 3. Data curation steps for individual data elements for BRAIN A-I

eTable 4. Standard vocabulary crosswalk for BRAIN-AI components

eTable 5. Temporal features and their associated definitions

eTable 6. Cohort ascertainment and exclusions for varied censored time horizons and feature windows at the development site

eTable 7. Predictive performance of the XGBoost model with a 12-hour censor horizon and 48-hour feature window in the validation dataset, after manual tuning, and after Bayesian tuning

eTable 8. Predictive performance of the XGBoost model with a 12-hour censor horizon and 48-hour feature window in the test dataset, after manual tuning, and after Bayesian tuning

eTable 9. Performance of the optimal models in the validation dataset at the development site

eTable 10. F1 scores of top performing models in the development site validation dataset

eTable 11. Fβ scores

eTable 12. Statistical performance of the 12-hour time horizon, 48-hour feature window XGBoost and logistic regression models

eTable 13. Cohort ascertainment and exclusions for varied feature windows for the validation site

© 2025 Horvat CM et al. JAMA Network Open.

eTable 14. Statistical performance of the extreme gradient boosting (XGBoost) and logistic regression generalizable models

eFigure 1. The process of BRAIN A-I model development and external validation

eFigure 2. A representation of the time window and censor horizons used to define cases and controls as part of the development, validation, and test cohorts

eFigure 3. Data cleaning and feature engineering process

eFigure 4. Plots of key statistical performance metrics

eFigure 5. Calibration plots and associated Brier scores for the top performing models for varied time horizons and features windows in the validation dataset

eFigure 6. Top 10 biomarker feature categories

eFigure 7. Plots of key statistical performance metrics for generalizable model performance

eFigure 8. Calibration plots for the generalizable model

eFigure 9. Feature importance analysis for the generalizable model

This supplemental material has been provided by the authors to give readers additional information about their work.

© 2025 Horvat CM et al. JAMA Network Open.

eMethods

1. Dataset Description

The development dataset contains patient encounters from the University of Pittsburgh Medical Center's (UPMC) Children's Hospital. These encounters are all from the hospital's quaternary pediatric intensive care unit (PICU) between January 1, 2010, and December 31, 2022. For the purposes of our study, we restrict our dataset to contain forty-five (45) biomarkers, Age (in months), and a Computational Outcome (described below). The 45 biomarkers are listed in eTables 4 and 5. The biomarkers include laboratory test results, vitals, medications, and non-laboratory diagnostic tests, which are all collected at irregular time intervals. Numerical biomarkers in our dataset are laboratory test results, vital signs, and non-laboratory diagnostic tests. The two categorical biomarkers are "Mechanical ventilator make/model" and "Pupillary Reaction". The Boolean biomarkers include ECMO Type, CRRT Therapy Type, whether the patient was on a ventilator, and medications.

2. Defining Case and Control Encounters

Cases at the development site were defined as patients who experienced a neurologic morbidity following initial admission to the pediatric intensive care unit (PICU) using the previously validated, computable, composite definition. Only encounters in which the admission time to outcome was longer than the specified censor horizon were included for prediction. If time from admission to start of the censor horizon was shorter than the feature window length, all data from admission up to the start of the censor window for prediction were included. This was done to maximize the number of included cases. Controls were defined as encounters that did not meet the definition of neurologic morbidity. Control encounters were included if the length of stay was longer than the specified feature window. A random feature window was selected from the entire encounter with preference given to a random window of time after the PICU admission if available. eFigure 1 provides a visual depiction of this cohort development process. At the development site, the neurologic morbidity outcome was a previously validated computable, composite definition. In summary, this consisted of the earliest occurrence of one of the following: 1) Order for brain magnetic resonance imaging; 2) Order for head computed tomography; 3) Order for electroencephalogram; 4) The earliest occurrence of either a behavioral medicine consult or an anti-delirium medication when both occurred within 72 hours of one another. Anti-delirium medications used at the study site include olanzapine, haloperidol, and dexmedetomidine. Encounters were dropped from the cohort ascertainment process if one of the following criteria were met: 1) insufficient data after applying the censor horizon; 2) insufficient data after applying the time window; 3) missing age, admission time, or discharge time; 4) discharge time occurred before the arrival time; 5) PICU length of stay <1 hour; 6) outcome occurred before the first PICU admission; 7) no SpO2 (pulse oximetry) measurement available for the encounter.

At the validation site, cases were defined as the placement of an order for a neurocritical care service consult. In contrast to the development site outcome, only the date and not the time of the validation site outcome was deemed accurate by investigators with knowledge of the local workflows. Accordingly, the outcome was defined with less precision compared to the outcome

© 2025 Horvat CM et al. JAMA Network Open.

used for the validation site. Similar to the development site, control encounters were those patients admitted to the PICU who did not have placement of a neurocritical care consult.

3. Data Cleaning and Feature Engineering

Raw clinical data are often not suited for use in many machine-learning algorithms. 1 Thus, data cleaning and feature engineering are required to convert the raw data into a format usable by machine learning algorithms.1 For example, erroneous and invalid data values must be corrected or removed, and categorical data values must be standardized. Furthermore, irregularly sampled temporal data and missing data values present challenging issues that must be addressed. Below, we describe how we addressed these issues in our data cleaning and feature engineering steps for non-temporal and temporal data. We then conclude with our approach to handling missing values. eFigure 2 provides a visual representation of this process.

3.1 Non-temporal Features

As the only non-temporal feature, age was binned into five groups: 'Under 1 Month', '1 to 12 Months', '12 to 144 Months', and 'Over 144 Months'; this is similar to other pediatric critical care risk scores 2.

3.2 Temporal Features

Irregularly sampled temporal data can be represented as non-temporal data by defining a fixed set of features that summarize the time series information (e.g., minimum or maximum value during a specified time window). However, before summarizing the temporal features, we must perform a data cleaning step to ensure the consistency and validity of the data in the features.

3.2.1 Numeric Temporal Features

Data Cleaning

Laboratory tests and vital sign values measured by more than one technique (e.g., invasive/non-invasive blood pressures) were grouped together, and names were standardized (e.g., "heart rate" and "pulse" were both standardized to "heart rate"). Numeric features containing text (e.g., a comment or result interpretation) or invalid characters (e.g., "<", ">") were extracted and then any remaining non-numeric values were removed. Then, duplicate entries were removed. After standardizing the feature values, we used Supplemental Table 4 as a guide to place boundaries for valid ranges of the numerical features.

Feature Engineering

We defined a time window (24 hours, 48 hours, or 72 hours) before the beginning of censor horizon, from which we extracted nineteen (19) non-temporal features for each numeric temporal feature. eTable 6 provides detailed definitions for the nineteen (19) different engineered features.

3.2.2 Categorical Temporal Features

Data Cleaning

© 2025 Horvat CM et al. JAMA Network Open.

We mapped each categorical value to a defined set of standard values. Mechanical ventilator names were standardized by removing punctuation and converting to lowercase. All duplicate entries were removed after standardizing the possible values for each categorical variable.

Feature Engineering

Using the same time window as for the numerical features, we engineered four (4) features to represent the ventilator make/model, and pupillary reaction features —first, last, second last, and mode.

3.2.3 Boolean Temporal Features

Data Cleaning

The Boolean features in our dataset were represented as 0 or 1. Therefore, like with the numeric features, we removed any text from the entries and extracted the numbers. Entries with a value outside of the range of 0 or 1 were ignored, and duplicates were dropped.

Feature Engineering

If the feature had a value of one (1 or True) at any point in the time window, then a single summarizing feature would be marked as one or True. Otherwise, they would be marked as False or 0.

3.2.4 Handling Missing Values

Some encounters had missing measurements required to compute each feature. Excluding encounters with incomplete data forfeits potentially valuable information. Therefore, the preferred approach to dealing with missing values is imputation 3. For Boolean features, missing values were imputed as 0 or False since missingness is usually due to the absence of an intervention or drug for the Boolean features in our dataset. However, for other types of data, imputation can hinder the interpretability of individual predictions, as it is usually unclear which values have been imputed and which are from the original dataset. Moreover, there is evidence that missing values can contain important predictive information.4,5 Therefore, instead of imputing, we opted to retain missingness in the numeric and categorical features to improve eventual model performance and interpretability. For categorical features, missing values were retained by simply adding a “missing” category. For numerical data, we first converted it to categorical data via discretization and added a “missing” category.4 Discretization is the process of converting continuous features or variables into discrete ones by dividing the continuous range into intervals. To discretize numerical features, we applied the “Minimum Description Length Principle Cut (MDLPC)” criterion algorithm. The MDLPC algorithm searches for the best cut-off and number of bins that maximize the feature’s information gain.6 After using this method to turn numerical data into categorical, we were able to add a ‘missing’ category/bin to numeric features.

After all these steps of feature engineering and processing, we ended up a set of 605 features which were derived from an initial set of 45 biomarkers (Supplemental Table 4) and Age.

© 2025 Horvat CM et al. JAMA Network Open.

4. Dataset Train/Test split and Hold-out

To learn and evaluate models at the development site, we split each of the approaches into a development dataset (data from 2010-2019) used for training (75% of the data from these years) and initial validation (25% of the data) of the models and a final hold-out dataset (data from 2020-2022) used to assess the performance of the most promising model from the development site. Per standard machine learning methodology, the training dataset was used to perform feature selection techniques and train models, the test dataset was used to choose the best models in development, and the hold-out dataset was used to evaluate the best-performing model.

4.1 Feature Selection

We examined two strategies for feature selection: 1) information gain (IG) filter with a threshold of 0, which results in selecting features that contain at least some predictive information for neurologic morbidity; 2) least absolute shrinkage and selection operator (LASSO), which is a form of penalized regression that shrinks certain coefficients to 0 to yield a reduced subset of the initial input variables as a final model. IG and LASOO feature selection was carried out using the Python package scikit-learn version 1.1.1, where scores were calculated by averaging the IG or LASSO score results from 10-fold cross-validation on the training dataset.

4.2 Model Learning

Since algorithm performance depends on the population of interest, feature measures, and outcomes being tested, it is generally a good idea to run multiple learning algorithms for any given prediction task.3,9 We, therefore, trained a Logistic Regression model, which is the standard model utilized in the clinical domain, in addition to three frequently utilized machine learning models—Random Forest, Naïve Bayes, and extreme gradient boosting (XGBoost), all coupled with the use of different feature selection methods and 10-fold cross-validation. Brief overviews of these algorithms can be found in Meyfroidt et al.9 All models were learned for each of the two training datasets using algorithm implementations provided in the Python package scikit-learn version 1.1.1, with the exception of XGBoost, which was implemented using XGBoost 1.7.3. Default algorithm settings were adopted for all algorithms except for the RF model, which was learned using 100 trees instead of the default of 10 trees.

5. Model Refinement

At the development site, the optimal model based on performance in the validation dataset was determined to be the XGBoost model with a 12-hour time horizon and 48-hour feature time-window. This model was further refined and tuned to promote explainability and optimized performance, which was then assessed in the holdout test dataset.

5.1 Feature refinement

Through the discretization process in our data wrangling pipeline, we ended up with features that contain unordered categorical values, which are ranges (“1 to 5”, “5 to 10”, etc.). To take advantage of the ordered ranking these feature values have, we turned these values into two

© 2025 Horvat CM et al. JAMA Network Open.

separate numbers (1, 5) and calculated their average. We then ranked the features by their average ((1+5)/2 = 3 -> rank = 0; (5+10)/2 = 7.5 -> rank = 1, etc.). 'Missing' values were assigned rank = -1.

A particular case was the 'Pupillary Reaction' feature, which did not contain a numeric range but rather labels of pupillary activity. We ranked these labels as follows: 0, normal; 1, one sluggish; 2, both sluggish; 3, one nonreactive; 4, both nonreactive. By turning these categories into numerical ranks, we can recover some of the ordinal information lost when the original numeric values were discretized into unordered categories.

5.2 Parameter Tuning

We performed two approaches to parameter tuning. The first was a manual approach where we selectively tuned the parameters of a single model. The parameters tuned were related to model complexity and model bias. In the second approach, we performed a search over all model parameters related to overfitting and class imbalance for all models. To do so, we used an optimization algorithm, which helped reduce the time it took to find suitable parameters for our dataset.

We only tuned based on the model performance on the validation dataset and did not tune based on the results of the test dataset. Also, our performance measure was based on the F1-Score metric implemented in Python's Scikit-Learn library. We only attempted to optimize the XGBoost 12-hour censor horizon and 48-hour time window model since it was the best performing model.

During manual parameter tuning we optimized the model's F1-Score on our validation dataset. To reduce the risk of overfitting, we actively reduced model complexity by capping the depth of the trees and how many trees we would use. For the maximum tree depth, we tried values in the range of 20 to 1; for the number of trees, we tried values in the range of 1 to 300. After various repetitions, we found that the depth and tree number combination that produced the highest F1-Score was 2 for depth (max_depth = 2) and 190 for the number of trees. To improve inference on the minority class (the samples with neurological morbidity), we adjusted weights on the positive class. We tried values in the range of 0 to 10 and, after various iterations, found the optimal weight was 2 (scale_pos_weight = 2).

For the large-scale parameter search we used the Bayesian optimization algorithm implemented in Python's Scikit-Optimize (version 0.8.1). We searched over all XGBoost parameters. We ran this algorithm for 500 iterations. In each iteration, a 10-fold cross-validation was performed, and the average F1-Score was calculated. After 500 iterations, the specifications for the parameters that resulted in the best F1-score were returned. After the manual and automated tuning, we observed improved model performance on the validation and test sets (eTables 7 and 8).

6. Development of a generalizable model

The development site model steps were repeated using a variable subset spanning 41 variables that were available at the external validation site to create a 'generalizable model' that could be

© 2025 Horvat CM et al. JAMA Network Open.

assessed using the local data at the external site. A 24-hour time horizon and 48-hour feature window model were selected after discussion with the external validation regarding what would be considered a clinically actionably time horizon knowing the model would be evaluated against an outcome of neurocritical care consult. A common data schema was shared with the external validation site along with data curation code. The XGBoost and logistic regression models performed comparably well at the development site and both were pickled (a process of serializing an object into a byte stream, which can then be transferred over a network) and sent to the external validation site for performance assessment.

7. Evaluation

Predictive performance of the models was evaluated using the test datasets. Model discrimination was assessed by calculating the area under the receiver operating characteristics curve (AUROC) and 95% confidence intervals (CIs). Predictive performance was also assessed by calculating the area under the precision-recall curve (AUPRC), which is an informative predictive measure that complements the AUROC for imbalanced datasets, i.e., datasets where the outcome of interest occurs rarely.10 Model calibration was assessed via inspection of observed versus predicted neurologic morbidity calibration belts and calculation of Brier scores. The optimal model was selected for assessment on the test dataset based on the F1 score, which is the harmonic mean of precision (positive predictive value) and recall (sensitivity), as well as cohort sizes, calibration, and data availability for eventual deployment. At the validation site, isotonic regression was applied in an effort to improve calibration of the best performing models as assessed by F1 scores.

To facilitate physician evaluation of models and elicit feedback, we utilized the Shapley additive explanations (SHAP) algorithm to generate explanations for the models.11,12 The SHAP algorithm is a model-agnostic approach to model interpretability, meaning that it is not tied to any specific model or learning algorithm and can thus be used to explain any model. The explanations generated are also relatively easy to interpret. The algorithm leverages concepts from game theory to calculate the average contribution of a feature towards a single prediction, which is called a SHAP value. Each SHAP value can be interpreted as the relative amount the feature is increasing or decreasing the prediction, with the relationship between the SHAP value and the change in prediction determined by the type of classifier approach (e.g., the SHAP value translates to a log-odds change in prediction for a tree-based method like XGBoost). A set of SHAP values forms an explanation for a single prediction. By generating SHAP explanations for many encounters and visualizing the aggregate SHAP values, we can get an overall idea of what each model learned.

To aid in understanding how the values of these biomarkers affect the model, we used the XAI algorithm, TreeSHAP, as implemented in Python's SHAP library.13 We used the white-box model created by TreeSHAP to calculate the feature importance for each patient in the hold-out set. Then, we calculated the average absolute feature importance over all the patients. Lastly, we estimated the importance of the 45 starting biomarkers by summing the average importance of all features constructed from each biomarker. SHAP explanations were generated using the SHAP library version 0.41.0 in Python with the training set as the background dataset.

© 2025 Horvat CM et al. JAMA Network Open.

Supplemental Methods References

1 Williams JB, Ghosh D, Wetzel RC. Applying Machine Learning to Pediatric Critical Care Data. *Pediatr Crit Care Med* 2018; **19**: 599–608.

2 Pollack MM, Holubkov R, Funai T, *et al.* The Pediatric Risk of Mortality Score: Update 2015. *Pediatr Crit Care Med* 2016; **17**: 2–9.

3 Awad A, Bader-El-Den M, McNicholas J, Briggs J. Early hospital mortality prediction of intensive care unit patients using an ensemble learning approach. *Int J Med Inform* 2017; **108**: 185–95.

4 Sharafoddini A, Dubin JA, Maslove DM, Lee J. A New Insight Into Missing Data in Intensive Care Unit Patient Profiles: Observational Study. *JMIR Med Inform* 2019; **7**: e11605.

5 López Pineda A, Ye Y, Visweswaran S, Cooper GF, Wagner MM, Tsui FR. Comparison of machine learning classifiers for influenza detection from emergency department free-text reports. *J Biomed Inform* 2015; **58**: 60–9.

6 "Multi-Interval Discretization of Continuous-Valued Attributes for Classification Learning" Usama M. Fayyad, Keki B. Irani. IJCAI 1993, Pro. of the Thirteenth International Joint Conference on Artificial Intelligence, Vol. 2, pp. 1022-1027, Morgan Kaufmann, Chambe'ry, France, August (1993). – Usama M. Fayyad, Ph.D. 1993; published online Aug 1. https://fayyad.com/multi-interval-discretization-of-continuous-valued-attributes-for-classification-learning-usama-m-fayyad-keki-b-irani-ijcai-1993-pro-of-the-thirteenth-international-joint-conference-on-artific/ (accessed Nov 2, 2023).

7 Delahanty RJ, Kaufman D, Jones SS. Development and Evaluation of an Automated Machine Learning Algorithm for In-Hospital Mortality Risk Adjustment Among Critical Care Patients. *Crit Care Med* 2018; **46**: e481–8.

8 Shickel B, Loftus TJ, Adhikari L, Ozrazgat-Baslanti T, Bihorac A, Rashidi P. DeepSOFA: A Continuous Acuity Score for Critically Ill Patients using Clinically Interpretable Deep Learning. *Sci Rep* 2019; **9**: 1879.

9 Meyfroidt G, Güiza F, Ramon J, Bruynooghe M. Machine learning techniques to examine large patient databases. *Best Pract Res Clin Anaesthesiol* 2009; **23**: 127–43.

10 Davis J, Goadrich M. The Relationship Between Precision-Recall and ROC Curves. University of Wisconsin-Madison Department of Computer Sciences, 2006 https://minds.wisconsin.edu/handle/1793/60482 (accessed Nov 2, 2023).

11 Lundberg S, Lee S-I. An unexpected unity among methods for interpreting model predictions. 2016; published online Dec 8. DOI:10.48550/arXiv.1611.07478.

12 Lundberg SM, Lee S-I. A Unified Approach to Interpreting Model Predictions. In: Advances in Neural Information Processing Systems. Curran Associates, Inc., 2017. https://papers.nips.cc/paper_files/paper/2017/hash/8a20a8621978632d76c43dfd28b67767-Abstract.html (accessed Nov 2, 2023).

© 2025 Horvat CM et al. JAMA Network Open.

13 Lundberg SM, Erion G, Chen H, *et al.* From local explanations to global understanding with explainable AI for trees. *Nat Mach Intell* 2020; **2**: 56–67.

© 2025 Horvat CM et al. JAMA Network Open.

Supplemental Biomarker Methods

Blood was obtained by a bedside nurse and set aside in collection tubes, which were then centrifuged at 4° Celsius and spun at 1500xg for 8 minutes. Serum was then retrieved, aliquoted and placed in storage at -80° Celsius. Enzyme-linked immunosorbent assays were used to measure serum levels of neuron specific enolase (NSE), myelin basic protein (MBP), and S100 calcium binding protein B, as previously described.[1,2] Hemolysis can interfere with measurement of NSE so a correction was applied using a validated formula.[3] Commercial assays for glial fibrillary acidic protein (GFAP), ubiquitin C-terminal hydrolase (UCH-L1), and alpha-II spectrin breakdown product 150 (SBDP150) were run by Banyan Biomarkers, Inc (San Diego, CA).

Supplemental Biomarker Methods References

1 Au AK, Bell MJ, Fink EL, Aneja RK, Kochanek PM, Clark RSB. Brain-Specific Serum Biomarkers Predict Neurological Morbidity in Diagnostically Diverse Pediatric Intensive Care Unit Patients. *Neurocrit Care* 2018; **28**: 26–34.

2 Raghu VK, Horvat CM, Kochanek PM, *et al.* Neurological Complications Acquired During Pediatric Critical Illness: Exploratory ‘Mixed Graphical Modeling’ Analysis Using Serum Biomarker Levels. *Pediatr Crit Care Med* 2021; published online June 1. DOI:10.1097/PCC.0000000000002776.

3 Berger R, Richichi R. Derivation and validation of an equation for adjustment of neuron-specific enolase concentrations in hemolyzed serum. *Pediatr Crit Care Med* 2009; **10**: 260–3.

© 2025 Horvat CM et al. JAMA Network Open.

eTable 1. Littenberg framework

**eTable 2.** Littenberg framework for the assessment of medical technology as applied to BRAIN A-I		
**Framework Domain**	**Description**	**Applicability to BRAIN A-I**
Biologic Plausibility	Does the current understanding of biology and disease pathology support the technology?	Practicing pediatric neurointensive care physicians posit that structured data including laboratory results, vital signs, medications, and other non-laboratory diagnostics can collectively be used to assess a child's risk of incurring or manifesting brain injury during the course of critical illness.
Technical Feasibility	Can the developed technology safely and reliably be delivered to the target patients?	Developing the model in adherance to the United States core data for interoperability and related informatics standard nomenclatures for structured data will facilitate model deployment.
Intermediate Outcomes	What are the biological, physiologic, or clinical effects of the technology?	During this development stage, model-calculated probabilities of neurologic deterioration were assessed for correlation with an available sample of measured, serum-based, brain-derived biomarkers.
Patient Outcomes	Are the intended patient outcomes promoted by use of the technology compatible with overall improved health?	The developed model is intended to alert clinicians but not prescribe a course of action, in large part owing to the complexity and heterogeneity of neurologic morbidity that occurs among critically ill children. Clinicians remain the ultimate arbiters of bedside decision-making that adequately accounts for the balance of risk and benefits related to a given management course.
Societal Outcomes	What are the external effects of the technology and does it confer benefit to the larger society?	Leveraging interoperability standards helps to reduce costs associated with technology deployment. By aiding clinicians in potentially obviating the occurrence or mitigating the effects of neurologic injury multiple population-level benefits are realized, including but not limited to a reduction in societal costs associated with long-term care of profound neurologic injury.

© 2025 Horvat CM et al. JAMA Network Open.

eTable 2.

**eTable 2**. Data curation steps for individual data elements for the BRAIN AI outcome.		
**File_Path**	**Outcome Marker**	**Type**
bh.csv	Behavioral Health Consult	behavioral
haldol.csv	Haloperidol	medication
olanzapine.csv	Olanzapine	medication
dexmedetomidine.csv	Dexmedetomidine	medication
eeg.csv	EEG	neuro
ct.csv	CT Head	neuro
mri.csv	MRI Brain	neuro

© 2025 Horvat CM et al. JAMA Network Open.

eTable 3.

eTable 3. Data curation steps for individual data elements for BRAIN A-I

Data Element	Min	Max	Action	Type	Available at Both Sites
Base deficit	-30	0	Discard	Numerical	Yes
Base excess	0	30	Discard	Numerical	Yes
Bicarbonate	0	80	Discard	Numerical	Yes
Blood urea nitrogen	0	200	Truncate	Numerical	Yes
Chloride	60	190	Discard	Numerical	Yes
Cisatracurium	0	1	Ignore	Boolean	Yes
C-Reactive Protein	0	100	Discard	Numerical	Yes
Creatinine	0.1	25	Discard	Numerical	Yes
CRRT Therapy Type	0	1	Discard	Boolean	No
DBP	0	200	Discard	Numerical	Yes
Dobutamine	0	1	Ignore	Boolean	Yes
Dopamine	0	1	Ignore	Boolean	Yes
ECMO Type	0	1	Discard	Boolean	No
Endotracheal tube	0	1	Discard	Boolean	No
Epinephrine	0	1	Ignore	Boolean	Yes
Fentanyl	0	1	Ignore	Boolean	Yes
Glucose	0	2000	Discard	Numerical	Yes
Hemoglobin	0	30	Discard	Numerical	Yes
Hydromorphone	0	1	Ignore	Boolean	Yes
INR	0	25	Discard	Numerical	No
Lactate	0	30	Discard	Numerical	Yes
Lorazepam	0	1	Ignore	Numerical	Yes
MBP	0	160	Discard	Numerical	Yes
Midazolam	0	1	Ignore	Boolean	Yes
Milrinone	0	1	Ignore	Boolean	Yes
Morphine	0	1	Ignore	Boolean	Yes
Norepinephrine	0	1	Ignore	Boolean	Yes
pCO2	5	150	Discard	Numerical	Yes
Peds Coma Score	3	15	Discard	Numerical	Yes
pH	6	8	Discard	Numerical	Yes
Platelets	0	5000	Discard	Numerical	Yes
Potassium	0.05	12	Discard	Numerical	Yes
Procalcitonin	0	250	Discard	Numerical	Yes
PTT	0	250	Truncate	Numerical	Yes
Pulse	0	350	Discard	Numerical	Yes
Pupillary Reaction				Categorical	Yes
Respiratory Rate	0	150	Discard	Numerical	Yes
SBP	0	300	Discard	Numerical	Yes

© 2025 Horvat CM et al. JAMA Network Open.

**eTable 3**. Data curation steps for individual data elements for BRAIN A-I					
Sodium	80	215	Discard	Numerical	Yes
SpO2	0	100	Discard	Numerical	Yes
Temperature	0	46	Discard	Numerical	Yes
Ventilated	0	1	Discard	Boolean	Yes
Ventilator Make/Model				Categorical	Yes
Weight	0	300	Discard	Numerical	Yes
White blood cell count	0	300	Discard	Numerical	Yes

© 2025 Horvat CM et al. JAMA Network Open.

eTable 4.

**eTable 4.** Standard vocabulary crosswalk for BRAIN-AI components					
**BRAIN A-I Components**	**Cerner Millennium Code Set**	**Standard Vocabulary**	**Standard Identifier**	**Standard Display**	**USCDI**
**Laboratory Tests**					
Base deficit	72 (Clinical Event Observation)	LOINC	1922-4	Base deficit in Arterial blood	Version 1
			1923-2	Base deficit in Capillary blood	
			1924-0	Base deficit in Venous blood	
			30318-0	Base deficit in Blood	
Base excess	72 (Clinical Event Observation)	LOINC	11555-0	Base excess in Blood by calculation	Version 1
			1925-7	Base excess in Arterial blood by calculation	
			1926-5	Base excess in Capillary blood by calculation	
			1927-3	Base excess in Venous blood by calculation	
Bicarbonate	72 (Clinical Event Observation)	LOINC	1959-6	Bicarbonate [Moles/volume] in Blood	Version 1
			1960-4	Bicarbonate [Moles/volume] in Arterial blood	
			1961-2	Bicarbonate [Moles/volume] in Capillary blood	
			14627-4	Bicarbonate [Moles/volume] in Venous blood	
			2028-9	Carbon dioxide, total [Moles/volume] in Serum or Plasma	
			20565-8	Carbon dioxide, total [Moles/volume] in Blood	
Blood urea nitrogen	72 (Clinical Event Observation)	LOINC	3094-0	Urea nitrogen [Mass/volume] in Serum or Plasma	Version 1
			6299-2	Urea nitrogen [Mass/volume] in Blood	
Chloride	72 (Clinical Event Observation)	LOINC	2075-0	Chloride [Moles/volume] in Serum or Plasma	Version 1
			2069-3	Chloride [Moles/volume] in Blood	
C-reactive protein	72 (Clinical Event Observation)	LOINC	1988-5	C reactive protein [Mass/volume] in Serum or Plasma	Version 1
Creatinine	72 (Clinical Event Observation)	LOINC	2160-0	Creatinine [Mass/volume] in Serum or Plasma	Version 1
			38483-4	Creatinine [Mass/volume] in Blood	
Glucose	72 (Clinical Event Observation)	LOINC	41653-7	Glucose [Mass/volume] in Capillary blood by Glucometer	Version 1
			2345-7	Glucose [Mass/volume] in Serum or Plasma	
			2339-0	Glucose [Mass/volume] in Blood	
Hemoglobin	72 (Clinical Event Observation)	LOINC	718-7	Hemoglobin [Mass/volume] in Blood	Version 1
			30351-1	Hemoglobin [Mass/volume] in Mixed venous blood	
			30313-1	Hemoglobin [Mass/volume] in Arterial blood	
			30350-3	Hemoglobin [Mass/volume] in Venous blood	
International normalized ratio	72 (Clinical Event Observation)	LOINC	6301-6	INR in Platelet poor plasma by Coagulation assay	Version 1
Lactate	72 (Clinical Event Observation)	LOINC	2519-7	Lactate [Moles/volume] in Venous blood	Version 1
			32693-4	Lactate [Moles/volume] in Blood	
Partial pressure of carbon dioxide	72 (Clinical Event Observation)	LOINC	2020-6	Carbon dioxide [Partial pressure] in Capillary blood	Version 1
			11557-6	Carbon dioxide [Partial pressure] in Blood	
			2019-8	Carbon dioxide [Partial pressure] in Arterial blood	
pH	72 (Clinical Event Observation)	LOINC	2745-8	pH of Capillary blood	Version 1
			11558-4	pH of Blood	
			2744-1	pH of Arterial blood	
			2746-6	pH of Venous blood	
Platelets	72 (Clinical Event Observation)	LOINC	777-3	Platelets [#/volume] in Blood by Automated count	Version 1

© 2025 Horvat CM et al. JAMA Network Open.

eTable 4. Standard vocabulary crosswalk for BRAIN-AI components					
**BRAIN A-I Components**	**Cerner Millennium Code Set**	**Standard Vocabulary**	**Standard Identifier**	**Standard Display**	**USCDI**
Potassium	72 (Clinical Event Observation)	LOINC	2823-3	Potassium [Moles/volume] in Serum or Plasma	Version 1
			6298-4	Potassium [Moles/volume] in Blood	
Procalcitonin	72 (Clinical Event Observation)	LOINC	33959-8	Procalcitonin [Mass/volume] in Serum or Plasma	Version 1
Partial thromboplastin time	72 (Clinical Event Observation)	LOINC	14979-9	aPTT in Platelet poor plasma by Coagulation assay	Version 1
Sodium	72 (Clinical Event Observation)	LOINC	2951-2	Sodium [Moles/volume] in Serum or Plasma	Version 1
			2947-0	Sodium [Moles/volume] in Blood	
White blood cell count	72 (Clinical Event Observation)	LOINC	6690-2	Leukocytes [#/volume] in Blood by Automated count	Version 1
			49498-9	Leukocytes [#/volume] in Blood by Estimate	
**Vital signs**					
Diastolic blood pressure	72 (Clinical Event Observation)	LOINC	8453-3	Diastolic blood pressure--sitting	Version 1
			8454-1	Diastolic blood pressure--standing	
			8455-8	Diastolic blood pressure--supine	
			8462-4	Diastolic blood pressure	
Glasgow coma scale score	72 (Clinical Event Observation)	LOINC	9269-2	Glasgow coma score total	Version 3
			9270-0	Glasgow coma score verbal	
			9267-6	Glasgow coma score eye opening	
			9268-4	Glasgow coma score motor	
Heart rate	72 (Clinical Event Observation)	LOINC	68999-2	Heart rate --supine	Version 1
			69000-8	Heart rate --sitting	
			69001-6	Heart rate --standing	
			8867-4	Heart rate	
			8890-6	Heart rate Cardiac apex by Auscultation	
Mean blood pressure	72 (Clinical Event Observation)	LOINC	8478-0	Mean blood pressure	Version 1
Pulse oximetry	72 (Clinical Event Observation)	LOINC	59408-5	Oxygen saturation in Arterial blood by Pulse oximetry	Version 1
Respiratory rate	72 (Clinical Event Observation)	LOINC	9279-1	Respiratory rate	Version 1
Systolic blood pressure	72 (Clinical Event Observation)	LOINC	8480-6	Systolic blood pressure	Version 1
Temperature	72 (Clinical Event Observation)	LOINC	60836-4	Esophageal temperature	Version 1
			76278-1	Bladder temperature via Foley	
			8310-5	Body temperature	
			8328-7	Axillary temperature	
			8329-5	Body temperature - Core	
			8331-1	Oral temperature	
			8332-9	Rectal temperature	
			8334-5	Body temperature – Urinary bladder	
Ventilator interface	72 (Clinical Event Observation)	LOINC	LL5542-7	Intubation tube types	Medical Device Class - Version Unknown
Ventilator make	72 (Clinical Event Observation)	LOINC	LL7706-7	Ventilator	Medical Device Class - Version Unknown
Weight	72 (Clinical Event Observation)	LOINC	29463-7	Body weight	Version 1
**Medications**					
Cisatracurium (Intravenous)	72 (Clinical Event Observation)	RxNorm RXCUI	319864	cisatracurium	Version 1
Dexmedetomidine (Intravenous)	72 (Clinical Event Observation)	RxNorm RXCUI	48937	dexmedeTOMIDine	Version 1

© 2025 Horvat CM et al. JAMA Network Open.

**eTable 4.** Standard vocabulary crosswalk for BRAIN-AI components					
**BRAIN A-I Components**	**Cerner Millennium Code Set**	**Standard Vocabulary**	**Standard Identifier**	**Standard Display**	**USCDI**
Dobutamine (Intravenous)	72 (Clinical Event Observation)	RxNorm RXCUI	3616	DOBUTamine	Version 1
Dopamine (Intravenous)	72 (Clinical Event Observation)	RxNorm RXCUI	3628	dopamine	Version 1
Epinephrine (Intravenous)	72 (Clinical Event Observation)	RxNorm RXCUI	3992	EPINEPHrine	Version 1
Fentanyl (Intravenous)	72 (Clinical Event Observation)	RxNorm RXCUI	4337	fentaNYL	Version 1
Haldol	72 (Clinical Event Observation)	RxNorm RXCUI	151839	Haldol	Version 1
Hydromorphone (Intravenous)	72 (Clinical Event Observation)	RxNorm RXCUI	3423	HYDROmorphone	Version 1
Lorazepam (Intravenous)	72 (Clinical Event Observation)	RxNorm RXCUI	6470	LORazepam	Version 1
Midazolam (Intravenous)	72 (Clinical Event Observation)	RxNorm RXCUI	6960	midazolam	Version 1
Milrinone (Intravenous)	72 (Clinical Event Observation)	RxNorm RXCUI	52769	milrinone	Version 1
Morphine (Intravenous)	72 (Clinical Event Observation)	RxNorm RXCUI	7052	morphine	Version 1
Norepinephrine (Intravenous)	72 (Clinical Event Observation)	RxNorm RXCUI	7512	norepinephrine	Version 1
Olanzapine	72 (Clinical Event Observation)	RxNorm RXCUI	61381	OLANZapine	Version 1
**Non-Laboratory Diagnostics**					
Brain computed tomography	72 (Clinical Event Observation)	LOINC	24725-4	CT Head	Version 2
Brain magnetic resonance imaging	72 (Clinical Event Observation)	LOINC	24590-2	MR Brain	Version 2
Electroencephalogram	72 (Clinical Event Observation)	LOINC	11523-8	EEG study	Version 2
Pupillary reaction	72 (Clinical Event Observation)	LOINC	79899-1	Left pupil Pupillary response	Observation Class – Version Unknown
		LOINC	79815-7	Right pupil Pupillary response	Observation Class – Version Unknown
**Consultation**					
Behavioral health consult	72 (Clinical Event Observation)	SNOMED CT	733870009	Assessment of delirium	Observation Class – Version Unknown
Abbreviations: aPTT, activated partial thromboplastin time; BRAIN A-I, biodigital rapid alert for identifying neuromorbidity A-I bundle; CT, computed tomography; EEG, electroencephalography; MR, magnetic resonance; LOINC, logical object identifiers, names, and codes; RXCUI, RxNorm concept unique identifier; SNOMED CT, systematized nomenclature of medicine - clinical terms; USCDI, United States Core Data for Interoperability					

© 2025 Horvat CM et al. JAMA Network Open.

eTable 5.

**eTable 5.** Temporal features and their associated definitions.		
#	Feature	Definition
1	Last	The last value prior to the censor window
2	Second to last	Second to last value prior to the censor window
3	First	First value prior to the censor window
4	Mean	Average of the values
5	Median	Median of the values
6	Minimum	Minimum of the values
7	Maximum	Maximum of the values
8	First slope	The first measured slope
9	Second to last slope	Second to last measured slope
10	Minimum slope	Minimum measured slope
11	Maximum slope	Maximum measured slope
12	% change second to last	% difference between the last and second to last value
13	% change first	% difference between the last and first values
14	% change minimum	% difference between the last and minimum values
15	% change maximum	% difference between the last and maximum values
16	First range	Difference between last and first values
17	Second to last range	Difference between the last and second to last value
18	Minimum range	Difference between the last and minimum values
19	Maximum range	Difference between the last and maximum values

© 2025 Horvat CM et al. JAMA Network Open.

eTable 6.

eTable6. Cohort ascertainment and exclusions for varied censored time horizons and feature windows at the development site.

Encounters with a PICU admission	**32,702**								
Feature window (hours)	24			48			72		
Censored time Horizon (hours)	6	12	24	6	12	24	6	12	24
Visit length or time to outcome hours less than feature window + time horizon	2925	3435	3908	7720	8230	8703	11593	12103	12576
Outcome occurred before PICU admission	4956								
Missing age, admission time, or discharge time	821								
PICU length of stay <1 hour	182								
No documented SpO_2 measurement	7								
Discharge time documented prior to admission time	2								
Final cohort	**23873**	**23363**	**22890**	**19078**	**18568**	**18095**	**15205**	**14695**	**14222**
Cases	**2841**	**2331**	**1858**	**2841**	**2331**	**1858**	**2841**	**2331**	**1858**
Controls	**21032**			**16237**			**12364**		

© 2025 Horvat CM et al. JAMA Network Open.

eTable 7.

eTable 7. Predictive performance of the XGBoost model with a 12-hour censor horizon and 48-hour feature window in the validation dataset, after manual tuning, and after Bayesian tuning.

	**XGBoost Base**	**XGBoost Manual Tuning**	**XGBoost Bayesian Tuning**
**AUROC**	0.84	0.84	0.85
**AUPRC**	0.61	0.62	0.63
**F1 Score**	0.54	0.56	0.58
**PPV**	0.77	0.75	0.63
**NPV**	0.92	0.92	0.93
**Sensitivity**	0.41	0.44	0.54
**Specificity**	0.98	0.98	0.95

Models were tuned to optimize the F1 score, bordered in bold.

Abbreviations: AUPRC, area under the precision recall curve; AUROC, area under the receiver operating characteristics curve; NPV, negative predictive value; PPV, positive predictive value; XGBoost, extreme gradient boosting.

© 2025 Horvat CM et al. JAMA Network Open.

eTable 8.

eTable 8. Predictive performance of the XGBoost model with a 12-hour censor horizon and 48-hour feature window in the test dataset, after manual tuning, and after Bayesian tuning.

	**XGBoost Base**	**XGBoost Manual Tuning**	**XGBoost Bayesian Tuning**
**AUROC**	0.87	0.88	0.89
**AUPRC**	0.66	0.68	0.69
**F1 Score**	0.57	0.58	0.62
**PPV**	0.86	0.79	0.74
**NPV**	0.93	0.93	0.94
**Sensitivity**	0.43	0.46	0.53
**Specificity**	0.99	0.98	0.98

Models were tuned to optimize the F1 score, bordered in bold.

Abbreviations: AUPRC, area under the precision recall curve; AUROC, area under the receiver operating characteristics curve; NPV, negative predictive value; PPV, positive predictive value; XGBoost, extreme gradient boosting.

© 2025 Horvat CM et al. JAMA Network Open.

eTable 9.

eTable 9. Performance of the optimal models in the validation dataset at the development site.

	Development Site								
**Feature Window (hours)**	**24**			**48**			**72**		
**Censored Time Horizon (hours)**	**6**	**12**	**24**	**6**	**12**	**24**	**6**	**12**	**24**
**Model**	LR	LR	LR	XGB	XGB	LR	XGB	LR	XGB
**Feature Selection**	IG	IG	IG	IG	IG	IG	IG	IG	IG
**AUROC**	0.83	0.83	0.80	0.87	0.82	0.82	0.88	0.86	0.82
**AUPRC**	0.59	0.49	0.39	0.73	0.61	0.53	0.78	0.71	0.61
**PPV**	0.74	0.65	0.60	0.82	0.79	0.69	0.88	0.75	0.68
**NPV**	0.92	0.92	0.93	0.92	0.92	0.93	0.91	0.92	0.92
**Sensitivity**	0.40	0.29	0.24	0.53	0.41	0.41	0.58	0.54	0.47
**Specificity**	0.98	0.98	0.99	0.98	0.98	0.98	0.98	0.97	0.97

Abbreviations: AUROC, area under the receiver operating characteristics curve; AUPRC, area under the precision recall curve; CFS, correlation-based feature selection; IG, information gain; LR, logistic regression; NB, naïve Bayes; NPV, negative predictive value; PPV, positive predictive value; XGB, XGBoost (extreme gradient boosting)

© 2025 Horvat CM et al. JAMA Network Open.

eTable 10.

eTable 10. F1 scores of top performing models in the development site validation dataset.

		Censored Time Horizon		
		**6-hours**	**12-hours**	**24-hours**
**Feature Window**	**24-hours**	LR<br>0.52	LR<br>0.40	LR<br>0.34
	**48-hours**	XGB<br>0.65	XGB<br>0.54	LR<br>0.51
	**72-hours**	XGB<br>0.70	LR<br>0.65	XGB<br>0.57

Cells denote the best performing model and the F1 score / Brier score.
All models at the development site were created using information gain feature selection
Abbreviations: LR, logistic regression; XGB, XGBoost (extreme gradient boosting)

© 2025 Horvat CM et al. JAMA Network Open.

eTable 11. Fβ scores

eTable 11A. F_β (β=2) scores of top performing models in the development site validation dataset.

		Censored Time Horizon		
		**6-hours**	**12-hours**	**24-hours**
**Feature Window**	**24-hours**	XGB 0.44 (0.44)	XGB 0.33 (0.32)	LR 0.27 (0.25)
	**48-hours**	LR 0.59 (0.58)	LR 0.49 (0.45)	LR 0.45 (0.39)
	**72-hours**	LR 0.66 (0.61)	LR 0.58 (0.55)	LR 0.51 (0.46)

Cells denote the best performing model and the F_β (β=2) score. XGB and LR were the top performing models. Values within each bracket are the F_β (β=2) scores of the other model.
All models at the development site were created using information gain feature selection.
Abbreviations: LR, logistic regression; XGB, XGBoost (extreme gradient boosting)

eTable 11B. F_β (β=3) scores of top performing models in the development site validation dataset.

		Censored Time Horizon		
		**6-hours**	**12-hours**	**24-hours**
**Feature Window**	**24-hours**	XGB 0.42 (0.42)	XGB 0.31 (0.30)	LR 0.26 (0.24)
	**48-hours**	LR 0.58 (0.55)	LR 0.48 (0.43)	LR 0.43 (0.37)
	**72-hours**	LR 0.64 (0.59)	LR 0.56 (0.53)	LR 0.50 (0.44)

Cells denote the best performing model and the F_β (β=3) score. XGB and LR were the top performing models. Values within each bracket are the F_β (β=3) scores of the other model. All models at the development site were created using information gain feature selection.
Abbreviations: LR, logistic regression; XGB, XGBoost (extreme gradient boosting)

eTable 11C. F_β (β=0.5) scores of top performing models in the development site validation dataset.

		Censored Time Horizon		
		**6-hours**	**12-hours**	**24-hours**
**Feature Window**	**24-hours**	XGB 0.62 (0.62)	XGB 0.52 (0.51)	LR 0.46 (0.46)
	**48-hours**	XGB 0.76 (0.72)	XGB 0.65 (0.64)	LR 0.61 (0.60)
	**72-hours**	XGB 0.79 (0.77)	XGB 0.73 (0.70)	XGB 0.67 (0.64)

Cells denote the best performing model and the F_β (β=0.5) score. XGB and LR were the top performing models. Values within each bracket are the F_β (β=0.5) scores of the other model.
All models at the development site were created using information gain feature selection.
Abbreviations: LR, logistic regression; XGB, XGBoost (extreme gradient boosting)

© 2025 Horvat CM et al. JAMA Network Open.

eTable 12. Statistical performance of the 12-hour time horizon, 48-hour feature window XGBoost and logistic regression models

eTable 12A. Statistical performance of the 12-hour time horizon, 48-hour feature window XGBoost and logistic regression models over a range of score thresholds in the development site validation dataset.

Threshold	AUROC	AUPRC	F1 Score	F2 Score	F3 Score	F0.5 Score	PPV	NPV	Sensitivity	Specificity	Confusion Matrix - (TN, FP, FN,TP)
**XGBoost Model**											
0.025	0.818	0.601	0.361	0.542	**0.650**	0.271	0.232	**0.956**	**0.813**	0.602	(1879, 1242, 86, 375)
0.05			0.422	**0.554**	0.619	0.341	0.302	0.945	0.701	0.761	(2375, 746, 138, 323)
0.1			0.483	**0.554**	0.582	0.429	0.399	0.938	0.614	0.863	(2694, 427, 178, 283)
0.3			**0.553**	0.508	0.494	0.607	0.649	0.926	0.482	0.962	(3001, 120, 239, 222)
0.5			0.536	0.454	0.432	0.654	0.766	0.919	0.412	0.981	(3063, 58, 271, 190)
0.7			0.508	0.411	0.386	**0.664**	0.836	0.913	0.364	0.989	(3088, 33, 293, 168)
0.9			0.429	0.325	0.301	0.629	**0.915**	0.904	0.280	**0.996**	(3109, 12, 332, 129)
**Logistic Regression Model**											
0.025	0.827	0.610	0.318	0.518	0.656	0.229	0.193	**0.966**	**0.894**	0.449	(1402, 1719, 49, 412)
0.05			0.384	0.561	**0.662**	0.292	0.252	0.958	0.809	0.645	(2012, 1109, 88, 373)
0.1			0.452	**0.574**	0.631	0.372	0.333	0.947	0.701	0.793	(2475, 646, 138, 323)
0.3			0.549	0.538	0.535	0.561	0.568	0.931	0.531	0.940	(2935, 186, 216, 245)
0.5			**0.557**	0.494	0.477	0.638	0.707	0.924	0.460	0.972	(3033, 88, 249, 212)
0.7			0.533	0.444	0.420	**0.668**	0.803	0.917	0.399	0.986	(3076, 45, 277, 184)
0.9			0.450	0.346	0.321	0.645	**0.908**	0.906	0.299	**0.996**	(3107, 14, 323, 138)

eTable 12B. Statistical performance of the 12-hour time horizon, 48-hour feature window XGBoost model over a range of score thresholds in the development site test dataset.

Threshold	AUROC	AUPRC	F1 Score	F2 Score	F3 Score	F0.5 Score	PPV	NPV	Sensitivity	Specificity	Confusion Matrix - (TN, FP, FN,TP)
**XGBoost Model**											
0.025	0.873	0.671	0.399	0.587	**0.698**	0.302	0.260	**0.974**	**0.859**	0.682	(2559, 1195, 69, 419)
0.05			0.493	0.621	0.679	0.409	0.367	0.962	0.750	0.832	(3124, 630, 122, 366)
0.1			0.573	**0.623**	0.642	0.531	0.505	0.954	0.662	0.916	(3438, 316, 165, 323)
0.3			**0.603**	0.538	0.519	0.685	0.754	0.938	0.502	0.979	(3674, 80, 243, 245)
0.5			0.562	0.463	0.437	**0.715**	0.874	0.929	0.414	0.992	(3725, 29, 286, 202)
0.7			0.491	0.383	0.357	0.684	0.926	0.920	0.334	0.997	(3741, 13, 325, 163)
0.9			0.390	0.287	0.264	0.610	**0.975**	0.910	0.244	**0.999**	(3751, 3, 369, 119)

© 2025 Horvat CM et al. JAMA Network Open.

eTable 13.

**eTable 13.** Cohort ascertainment and exclusions for varied feature windows for the validation site.	
Timeframe	**4/2018-2023**
Encounters with a PICU admission	**9,039**
Feature Window	48-hours
Visit length <24 hours or PICU length of stay <1 hour	1,791
Missing age, discharge time or sex	329
No documented SpO_2 measurement	15
No accurate Neuro Consultation records prior 2018	---
Outcome event prior to PICU admission	79
Key data missing within Feature Window	---
Final Cohort	**6,825**
Cases	**387**
Controls	**6,438**

© 2025 Horvat CM et al. JAMA Network Open.

eTable 14. Statistical performance of the extreme gradient boosting (XGBoost) and logistic regression generalizable models

eTable 14A. Statistical performance of the extreme gradient boosting (XGBoost) and logistic regression generalizable models at the development site across varied output thresholds.

Threshold	AUROC	AUPRC	F1 Score	F2 Score	F3 Score	F0.5 Score	PPV	NPV	Sensitivity	Specificity	Confusion Matrix - (TN, FP, FN,TP)
**XGBoost Model**											
0.025	0.872	0.617	0.365	0.551	0.665	0.272	0.233	0.976	0.837	0.708	(2658, 1096, 65, 333)
0.05			0.459	0.598	0.666	0.372	0.330	0.970	0.751	0.838	(3147, 607, 99, 299)
0.1			0.528	0.591	0.615	0.478	0.450	0.960	0.641	0.917	(3442, 312, 143, 255)
0.3			0.547	0.481	0.462	0.635	0.711	0.943	0.445	0.981	(3682, 72, 221, 177)
0.5			0.489	0.394	0.370	0.645	0.818	0.935	0.349	0.992	(3723, 31, 259, 139)
0.7			0.421	0.316	0.292	0.629	0.939	0.928	0.271	0.998	(3747, 7, 290, 108)
0.9			0.312	0.222	0.202	0.527	0.974	0.921	0.186	0.999	(3752, 2, 324, 74)
**Logistic Regression Model**											
0.025	0.855	0.605	0.303	0.497	0.633	0.218	0.183	0.977	0.869	0.590	(2213, 1541, 52, 346)
0.05			0.388	0.552	0.643	0.299	0.260	0.969	0.769	0.767	(2881, 873, 92, 306)
0.1			0.481	0.571	0.609	0.415	0.380	0.960	0.653	0.887	(3330, 424, 138, 260)
0.3			0.562	0.513	0.499	0.621	0.668	0.947	0.485	0.974	(3658, 96, 205, 193)
0.5			0.520	0.429	0.406	0.658	0.801	0.938	0.384	0.990	(3716, 38, 245, 153)
0.7			0.489	0.383	0.357	0.677	0.911	0.934	0.334	0.997	(3741, 13, 265, 133)
0.9			0.367	0.267	0.245	0.584	0.968	0.924	0.226	0.999	(3751, 3, 308, 90)

© 2025 Horvat CM et al. JAMA Network Open.

eTable 13B. Statistical performance of the extreme gradient boosting (XGBoost) and logistic regression generalizable models at the external validation site across varied output thresholds.

Threshold	AUROC	AUPRC	F1 Score	F2 Score	F3 Score	F0.5 Score	PPV	NPV	Sensitivity	Specificity	Confusion Matrix - (TN, FP, FN,TP)
**XGBoost Model**											
0.025	0.809	0.509	0.120	0.253	0.401	0.079	0.064	0.986	0.964	0.155	(1001, 5437, 14, 373)
0.05			0.136	0.279	0.430	0.090	0.073	0.987	0.935	0.290	(1869, 4569, 25, 362)
0.1			0.158	0.308	0.450	0.106	0.087	0.980	0.840	0.471	(3030, 3408, 62, 325)
0.3			0.255	0.410	0.515	0.185	0.157	0.977	0.690	0.777	(5000, 1438, 120, 267)
0.5			0.368	0.485	0.542	0.297	0.263	0.975	0.615	0.896	(5770, 668, 149, 238)
0.7			0.477	0.509	0.520	0.449	0.432	0.971	0.532	0.958	(6167, 271, 181, 206)
0.9			0.480	0.403	0.383	0.594	0.705	0.963	0.364	0.991	(6379, 59, 246, 141)
**Logistic Regression Model**											
0.025	0.815	0.478	0.112	0.239	0.384	0.073	0.060	0.980	0.974	0.075	(485, 5953, 10, 377)
0.05			0.121	0.255	0.402	0.080	0.065	0.985	0.956	0.170	(1097, 5341, 17, 370)
0.1			0.139	0.281	0.428	0.092	0.075	0.982	0.897	0.336	(2162, 4276, 40, 347)
0.3			0.216	0.384	0.518	0.151	0.125	0.982	0.796	0.666	(4285, 2153, 79, 308)
0.5			0.293	0.451	0.549	0.217	0.185	0.979	0.703	0.814	(5240, 1198, 115, 272)
0.7			0.374	0.478	0.527	0.307	0.274	0.973	0.587	0.907	(5837, 601, 160, 227)
0.9			0.487	0.469	0.463	0.507	0.521	0.968	0.457	0.975	(6275, 163, 210, 177)

© 2025 Horvat CM et al. JAMA Network Open.

eFigure 1.

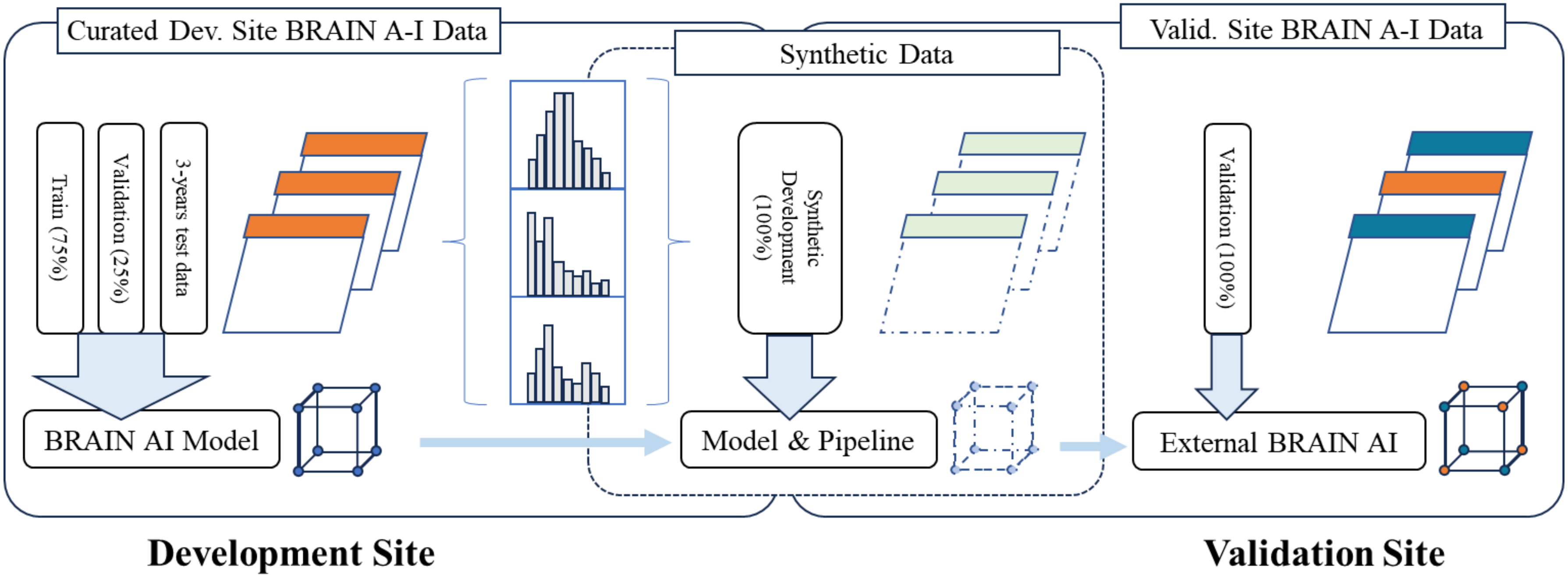

eFigure 1. The process of BRAIN A-I model development and external validation. Curated data at the development site were divided into a train cohort, validation cohort, and 2-years of holdout test data. The curated data were used to generate synthetic data with comparable single variable statistical distributions. The synthetic data were then distributed to the external validation site with the generalizable model, facilitating local data curation by providing the necessary details of data structure. Finally, the working BRAIN A-I model and pipeline were applied to real-world data at the external validation site. Abbreviations: BRAIN A-I, Biodigital Rapid Alert to Identify Neuromorbidity A-I Bundle; Dev. Site, Development Site; Valid. Site, Validation Site.

© 2025 Horvat CM et al. JAMA Network Open.

eFigure 2.

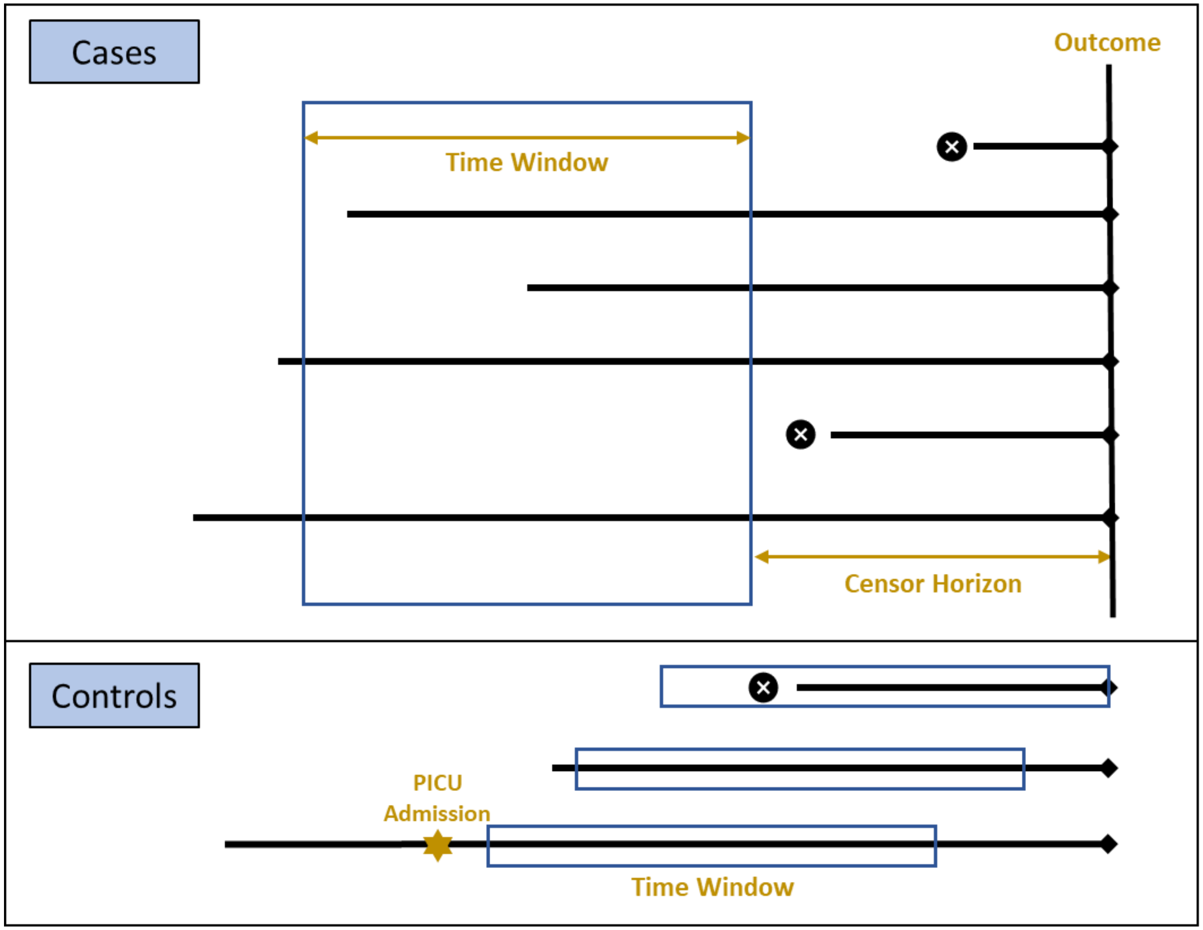

eFigure 2. A representation of the time window and censor horizons used to define cases and controls as part of the development, validation, and test cohorts. The blue boxes in the top, ‘Cases’ box identify a time window that is also demarcated by a horizontal, gold, bidirectional arrow, the horizontal black lines represent length of stay for individual encounters, the vertical black line represents the occurrence of the neurological morbidity outcome, and the gold, horizontal, bidirectional arrow indicates the censor horizon, or period of time that data were not incorporated into the model. In the bottom, ‘Controls’ box, the blue boxes indicate the time window of data used for each stage of model development and evaluation. The white ‘x’s’ in black circles indicate the start of an encounter. The plot is a composite representation of a variety of patient scenarios. All case windows occurred during the PICU admission, while priority was given to windows after the start of a PICU admission for controls if available.

© 2025 Horvat CM et al. JAMA Network Open.

eFigure 3.

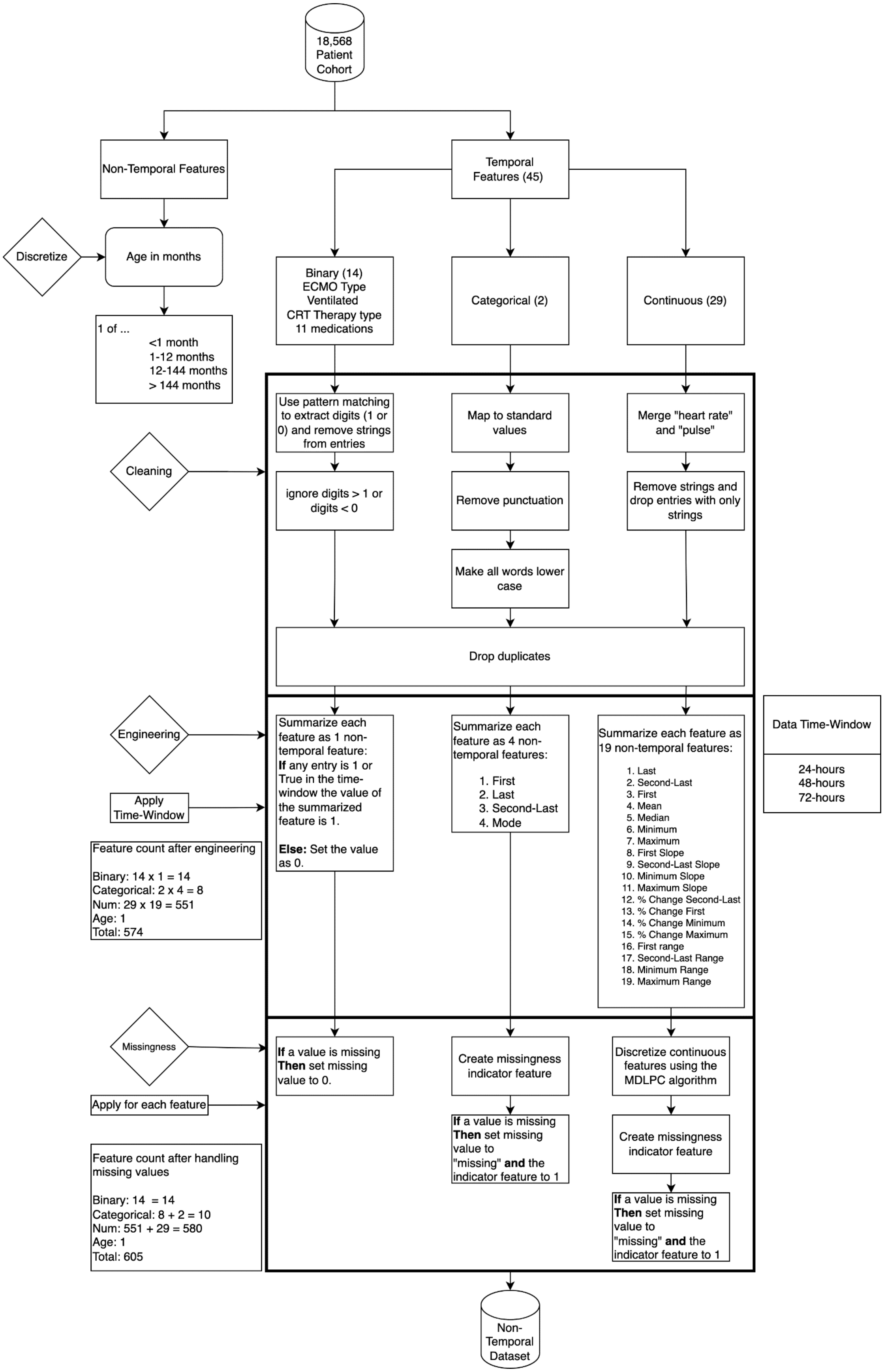

eFigure 3. Data cleaning and feature engineering process.

© 2025 Horvat CM et al. JAMA Network Open.

eFigure 4.

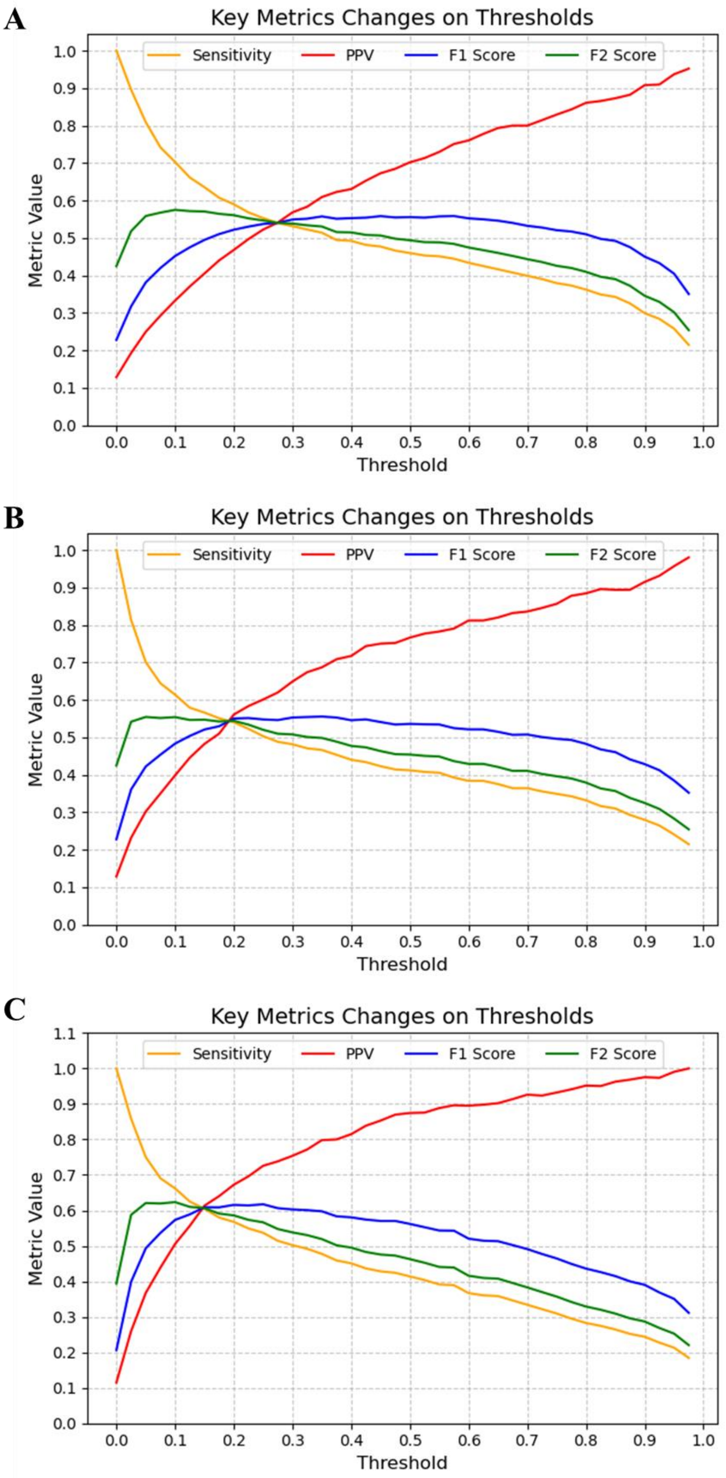

eFigure 4. Plots of the key statistical performance metrics sensitivity (gold line), positive predictive value (PPV, red line), F1 score (blue line), and F2 score (green line) with metric values on the y-axis and model output thresholds on the x-axis for A) the logistic regression model with a 12-hour time horizon and 48-hour feature window in the development site validation dataset; B) the extreme gradient boosting model with a 12-hour time horizon and 48-hour feature window in the development site validation dataset; and C) the extreme gradient boosting model with a 12-hour time horizon and 48-hour feature window in the development site test dataset.

© 2025 Horvat CM et al. JAMA Network Open.

eFigure 5.

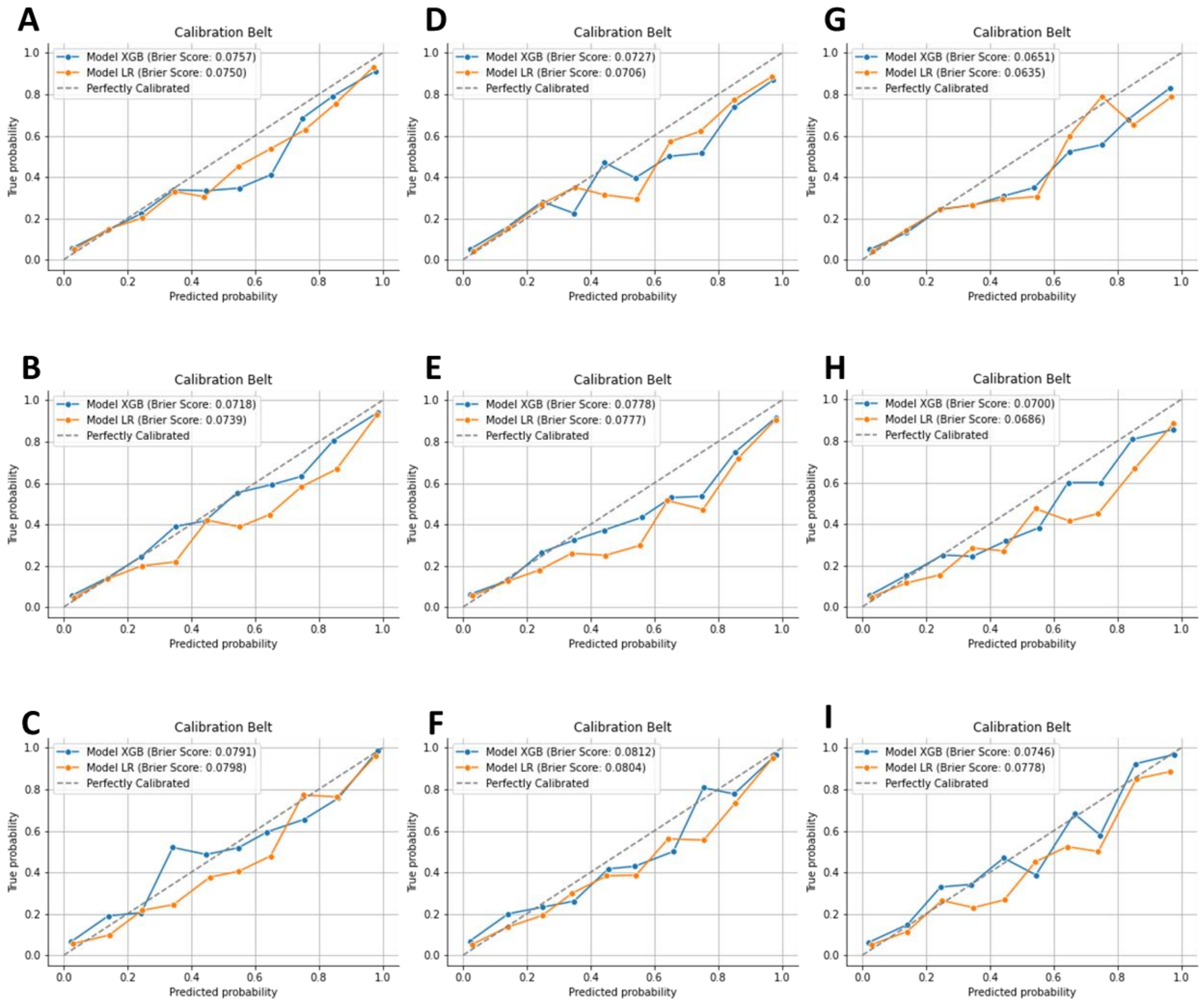

eFigure 5. Calibration plots and associated Brier scores for the top performing models for varied time horizons and features windows in the validation dataset. Calibration was overall excellent with residual small bias towards overprediction. A) 6-hour time horizon and 24-hour feature window; B) 6-hour time horizon and 48-hour feature window; C) 6-hour time horizon and 72-hour feature window; D) 12-hour time horizon and 24-hour feature window; E) 12-hour time horizon and 48-hour feature window; F) 12-hour time horizon and 72-hour feature window; G) 24-hour time horizon and 24-hour feature window; H) 24-hour time horizon and 48-hour feature window; I) 24-hour time horizon and 72-hour feature window. Abbreviations: LR, logistic regression; XGB, extreme gradient boosted.

© 2025 Horvat CM et al. JAMA Network Open.

eFigure 6.

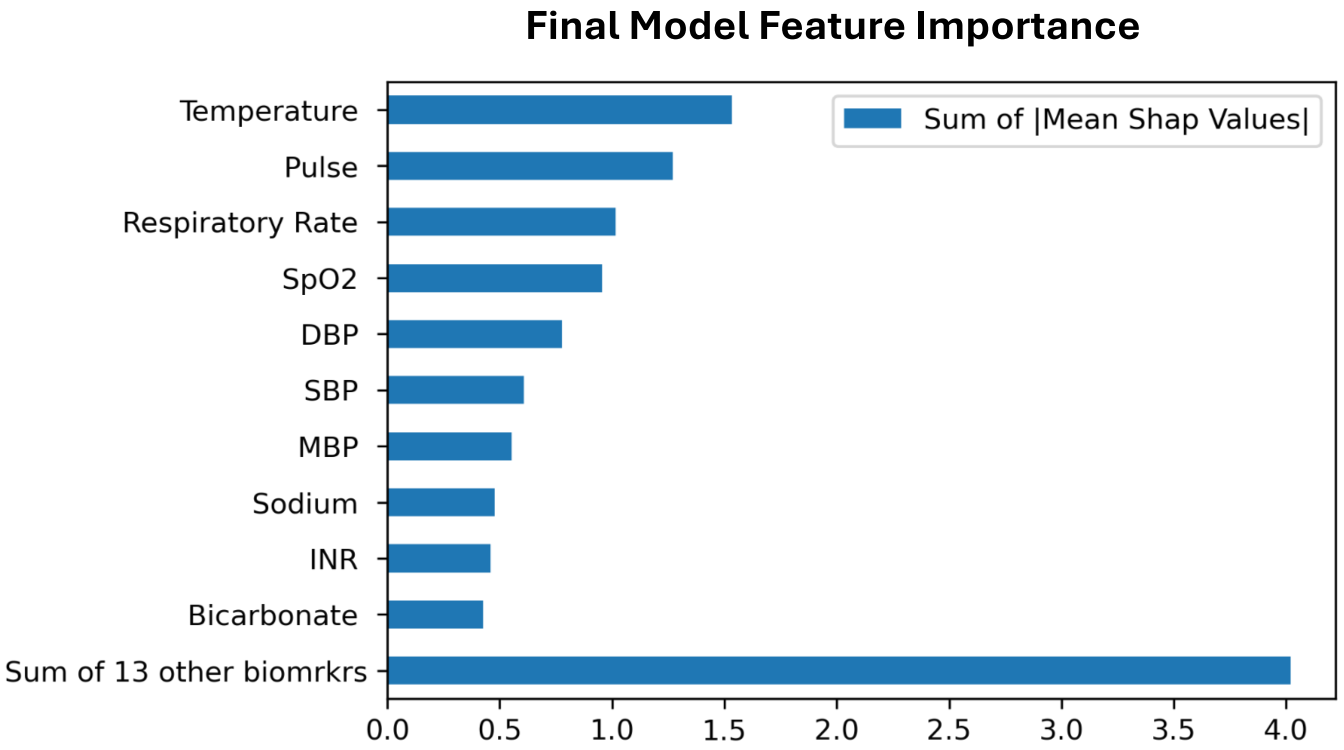

eFigure 6. Top 10 biomarker feature categories based on Shap values for the 12-hour time horizon 48-hour feature window XGBoost model from the development site. Each category contains several features, e.g. Temperature contains maximum temperature, minimum temperature, average temperature, etc. The blue bars represent the sum of the mean absolute Shap values for each category of features. Abbreviations: biomrkrs, biomarkers.

© 2025 Horvat CM et al. JAMA Network Open.

eFigure 7.

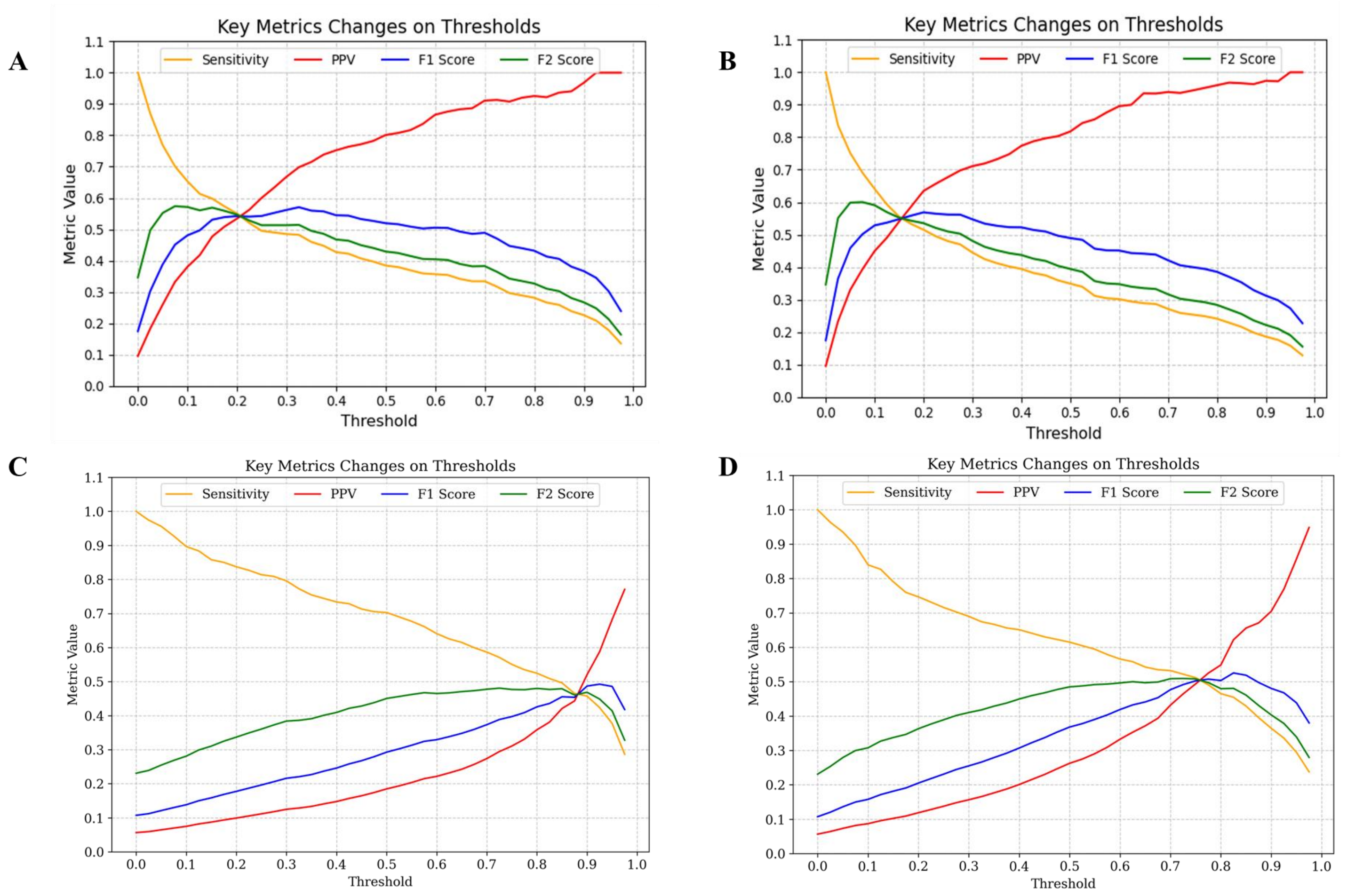

eFigure 7. Plots of the key statistical performance metrics sensitivity (gold line), positive predictive value (PPV, red line), F1 score (blue line), and F2 score (green line) with metric values on the y-axis and model output thresholds on the x-axis for the generalizable A) logistic regression model in the development site test dataset; B) extreme gradient boosting model in the development site test data set; C) logistic regression model in the external validation dataset; D) extreme gradient boosting model in the external validation site dataset.

© 2025 Horvat CM et al. JAMA Network Open.

eFigure 8.

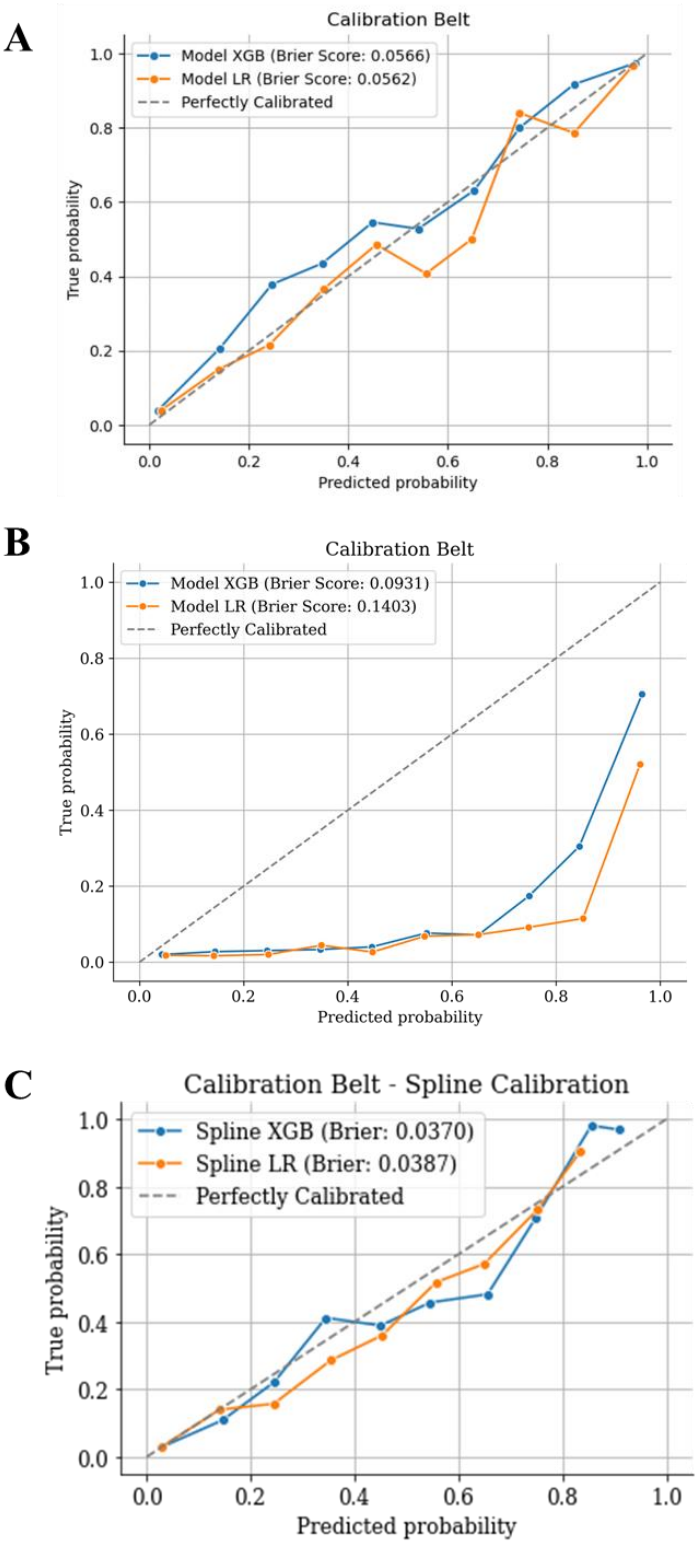

eFigure 8. Calibration plots for the generalizable model in the A) development site test dataset, B) the external validation site dataset, and C) the external validation site dataset after spline recalibration.

© 2025 Horvat CM et al. JAMA Network Open.

eFigure 9.

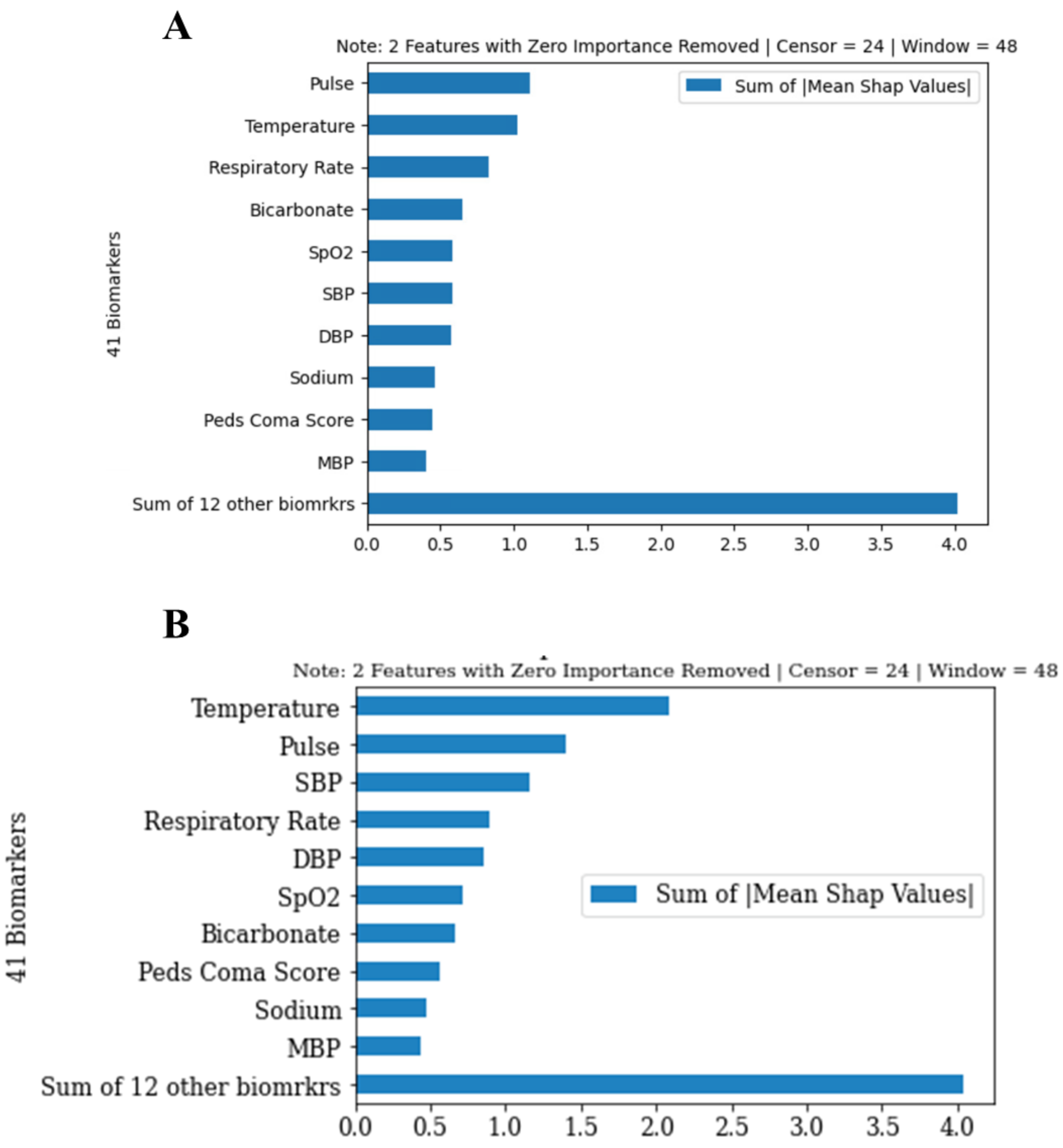

eFigure 9. Feature importance analysis for the generalizable model in the A) development site test dataset and B) external validation site dataset.
