# Supplementary material for: Interoperable Models for Identifying Critically Ill Children at Risk of Neurologic Morbidity
Source: JAMA Netw Open. 2025 Feb 4;8(2):e2457469. doi: 10.1001/jamanetworkopen.2024.57469 (PMC11795326; doi:10.1001/jamanetworkopen.2024.57469)
Supplement: Supplement 2. — Data Sharing Statement [file jamanetwopen-e2457469-s002.pdf]

Data Sharing Statement

Horvat. Interoperable Models for Identifying Critically Ill Children at Risk of Neurologic Morbidity. *JAMA Netw Open*. Published February 04, 2025.
doi:10.1001/jamanetworkopen.2024.57469

Data
Data available: No

Additional Information
Explanation for why data not available: Data will be available on request for specific use cases.
